# OSMAC guided bioprospecting of Atlantic sponges reveals novel bacterial species and evidence for the antimicrobial thiazole alkaloid agrochelin II

**DOI:** 10.1128/aem.01877-25

**Published:** 2026-02-17

**Authors:** Sam E. Williams, Henry L. Stennett, Andrew J. Devine, Zhongshu Song, Catherine R. Back, Luoyi Wang, Judith Mantell, Chris Neal, Mark A. Jepson, Angela E. Essex-Lopresti, Jonathan D. Sellars, James E. M. Stach, Christine L. Willis, Paul Curnow, Paul R. Race

**Affiliations:** 1 School of Biochemistry, University of Bristol 152328, Bristol, United Kingdom; 2 Novo Nordisk Foundation Center for Biosustainability, Technical University of Denmark 587234, Lyngby, Denmark; 3 School of Chemistry, University of Bristol 152332, Bristol, United Kingdom; 4 Institute of Microbiology, Chinese Academy of Sciences 85387, Beijing, China; 5 Wolfson Bioimaging Facility, University of Bristoly 630016, Bristol, United Kingdom; 6 Defence Science and Technology Laboratory 13330, Salisbury, United Kingdom; 7 School of Pharmacy, Newcastle University 573583, Newcastle upon Tyne, United Kingdom; 8 School of Natural and Environmental Sciences, Newcastle University 98458, Newcastle upon Tyne, United Kingdom; Norwegian University of Life Sciences, Ås, Norway

**Keywords:** antibiotics, deep-sea, natural products, microbiome, metabolomics, OSMAC, genome mining, siderophores

## Abstract

The deep sea remains a major reservoir of underexplored microbial diversity and biosynthetic novelty. Here, we describe three bacterial species isolated from Atlantic sponges, including *Stappia quadratibracata* sp. nov., *Bacillus crepusculi* sp. nov., and *Psychrobacter noctis* sp. nov. Genome sequencing, phylogenomics, and phenotypic characterization confirmed their novelty and biosynthetic potential. A targeted ‘One Strain Many Active Compounds’ (OSMAC) screen revealed previously silent antibacterial activity from *Stappia quadratibracata* sp. nov., only when grown with the carbon source succinate. Metabolomics and molecular networking analysis indicated that this activity was attributable to an unstable thiazole alkaloid with spectral data closely related to, but distinct from, the epimeric siderophores agrochelin and massiliachelin. Spectroscopic studies, in tandem with comparative analysis of the biosynthetic gene cluster for this metabolite, are consistent with agrochelin II, a previously unreported thiazole alkaloid diastereoisomer. Agrochelin II exhibits iron-enhanced antibacterial activity against *Staphylococcus aureus,* underscoring the ecological role of iron acquisition in microbial competition. Our findings highlight the value of OSMAC-guided bioprospecting in uncovering antimicrobial metabolites from sponge-associated bacteria.

**IMPORTANCE:**

Bioactive microbial natural products remain the preeminent source of new lead compounds for drug development. Due to the increasingly high levels of strain and compound rediscovery from terrestrial environments, the deep-ocean is increasingly considered an attractive starting point for bioprospecting programs, which seek to isolate and characterize novel chemical scaffolds. Here, we use a combination of genomics, metabolomics, and chemical analysis, to establish the biosynthetic potential of three bacterial species isolated from deep-ocean Atlantic sponges and report the discovery and characterization of a new antimicrobial thiazole alkaloid, agrochelin II. Our findings demonstrate the usefulness of integrated cultivation–screening-metabolomics–genomics pipelines for microbial metabolite discovery and identify the genus *Stappia* as a hitherto neglected source of bioactive natural products.

## INTRODUCTION

Underexplored environments such as the deep ocean, caves, and hot springs have been suggested as potential sources of microorganisms that produce new antibiotics (1–3). The conditions of these environments are inhospitable to most life forms, with microbial occupants of these niches frequently possessing metabolic innovations that promote resilience (4–6). These innovations may include the ability to biosynthesize natural products with novel structures and functions (7, 8). Marine organisms have historically yielded clinically important natural products, emphasizing their continued potential in drug discovery (9–11). Bull et al. posited that chemical diversity follows from biological diversity, thus by characterizing new species, we increase the probability of discovering new antimicrobial natural products (12). Broadening bioprospecting beyond well characterized gifted taxa, e.g., the actinomycetes, may also help to identify previously overlooked microorganisms and their associated bioactive metabolites (13, 14).

Accessing the full spectrum of chemical diversity from environmental strains is an ongoing challenge in natural product discovery (15, 16). The “One Strain Many Active Compounds” (OSMAC) approach is an experimental strategy that can be employed to identify the chemical diversity produced by a single strain (17). In this method, the composition of media or carbon source is adjusted in an effort to activate biosynthetic gene clusters (BGCs) that are not expressed under standard laboratory conditions. Importantly, this approach has proven to be of particular value in the identification of antimicrobial natural products (17). By applying this method to underexplored bacterial taxa, it may be possible to expedite the discovery of new antimicrobial natural products, by leveraging the additive benefits of targeting historically neglected microbes using OSMAC.

As one of Earth’s largest biomes, the deep-sea and abyssal oceanic zones encompass depths beyond ~200 m and feature low temperatures, high-pressure, oligotrophic and dark conditions (18). These unique conditions have led to specialized metabolic adaptations in deep-sea microbial communities, providing an underexplored and distinct genetic resource for biodiscovery (19, 20). As part of an ongoing antibiotic discovery program, we have isolated hundreds of bacterial strains from deep-sea sponges (21). The sponges that comprise the “Bristol Sponge Microbiome Collection” (BISECT) were recovered from an area of the equatorial Atlantic not previously prospected, at depths of up to 3 km and comprising diverse microbial communities (21–23). Although the average depth of the ocean is 1.2 km (24), most sponge bioprospecting studies have to date sampled tropical waters of less than 200 m deep, a consequence of the ease of sample recovery from these locations (25). In contrast, at least two-thirds of the BISECT sponges were collected at depths exceeding 1 km (21). It has been widely noted that the extreme conditions of pressure, temperature, and salinity found in deep-sea habitats have driven the evolution of diverse and unusual bacteria, which constitute intriguing targets for the discovery of new natural products (5, 26, 27). To this end, we have been engaged in a taxonomic identification of culturable bacteria from the BISECT collection.

Herein, we describe three culturable strains isolated from BISECT sponges and propose the names *Stappia quadratibracata* sp. nov., *Bacillus crepusculi* sp. nov., and *Psychrobacter noctis* sp. nov. We have examined the biosynthetic potential of these bacteria and by systematically adjusting culture conditions activated previously silent anti-methicillin-resistant *Staphylococcus aureus* (MRSA) activity in *S. quadratibracata*. This activity is shown to be attributable to the previously unreported thiazole alkaloid agrochelin II, whose chemical structure has been elucidated using an approach combining spectroscopic studies with bioinformatic analysis. Collectively, our findings demonstrate the usefulness of employing OSMAC to unlock the biosynthetic potential of deep-sea bacteria, including those from less well-studied genera.

## RESULTS

### Isolation and ecology

Three bacterial strains were isolated from individual deep-sea sponges: 28M-7^T^ from sponge B0078, 28A-2^T^ from B01641, and 28M-43^T^ from B01661. Sponges were collected at depths between 868 and 1,483 m in the twilight (mesopelagic, 150–1,000 m) and midnight (bathypelagic, 1,000–4,000 m) zones of the equatorial Atlantic at the Vayda and Knipovich seamounts (28). Seawater samples were analyzed at seven sites (1,150–2,307 m depth) on the seamounts and ranged between 34.93–35.00 practical salinity units, 165.77–236.25 μmol/kg oxygen, and 2.80–5.23°C. The data describing each of these sponges are presented in Table 1.

**TABLE 1:** Characteristics of the sponges from which the bacteria described in this study were isolated^*a*^

Sponge	Class	Depth (m)	Region	Coordinates
B0078	Demosponge	971	Knipovich	5° 37' 30.0" N 26° 57' 29.0" W
B01641	Hexactinellid	868	Vayda	14° 53' 03.0" N 48° 07' 27.0" W
B01661	Demosponge	1,483	Vayda	14° 51' 47.0" N 48° 14' 29.0" W

^*a*^ The class of each sponge was estimated based on its morphology. The depths, region, and coordinates shown are those at which each sponge was collected from the sea floor.

### Phylogeny

We sequenced the genomes of the three strains using a hybrid approach (Illumina and Oxford Nanopore) and generated high-quality, biologically accurate assemblies (Table S1). To resolve their taxonomic novelty, we conducted comprehensive genomic comparisons including digital DNA-DNA hybridization (dDDH), average nucleotide identity (ANI), and GTDB-based phylogenomic placement (29–33).

#### Strain 28M-7^T^

**Fig 1:**
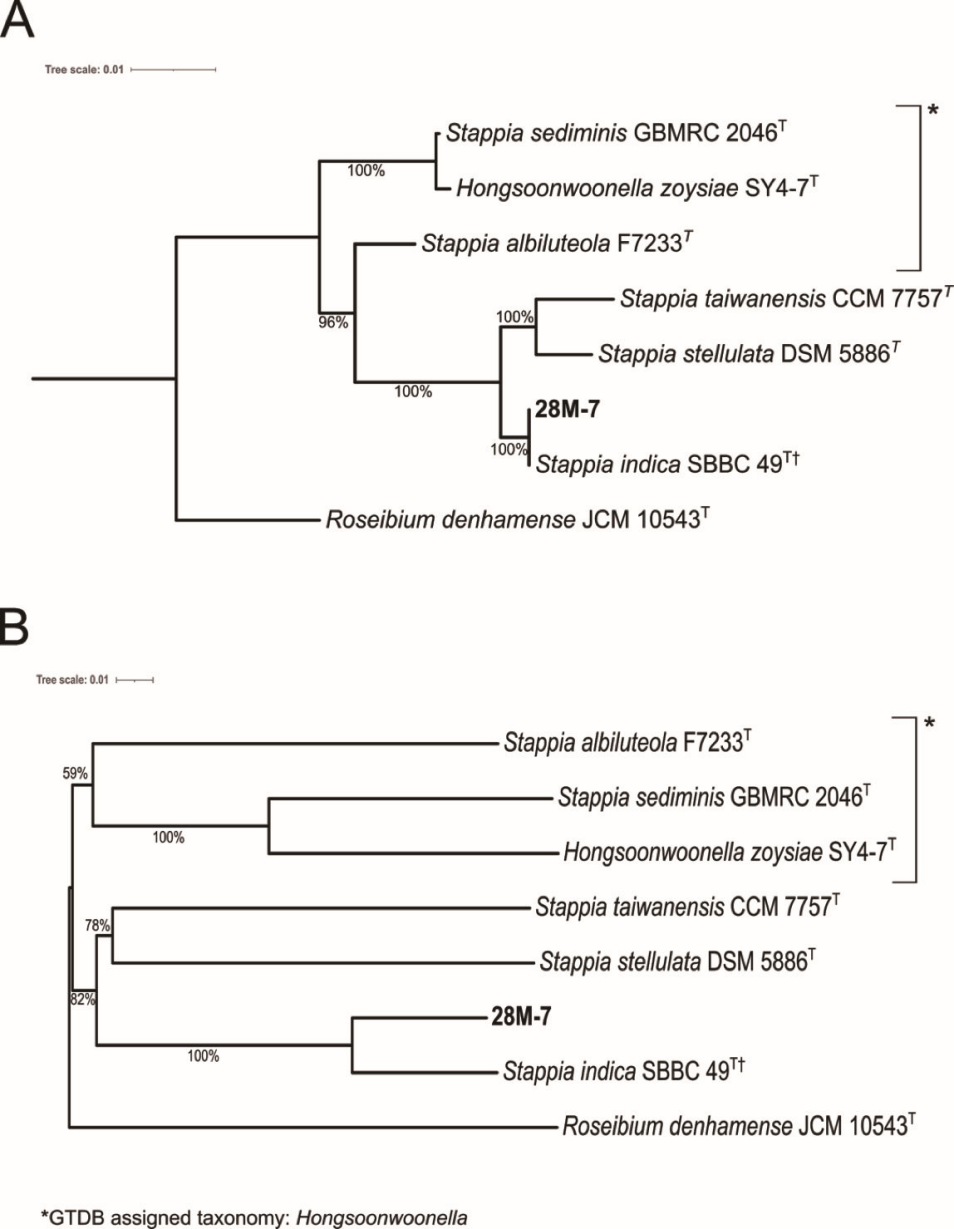
(**A**) 16S rRNA gene-based phylogenetic tree of 28M-7^T^. (**B**) Whole genome phylogenetic tree for strain 28M-7^T^ which we propose as *Stappia quadratibracata* sp. nov. Trees were constructed with TYGS (31) and prepared using iTOL (39). GBDP pseudo-bootstrap values from 100 replications are shown, bootstrap values below 50% are not shown. Branch lengths are scaled in terms of GBDP d5 distance formula. The trees were rooted using *Roseibium denhamense* JCM 10543^T^, another member of the family Stappiaceae, as the outgroup. ^†^*Stappia indica* SBBC 49 included as GTDB species representative as no genome is available for the type strain *S. indica* B106^T^. The whole genome tree had an average branch support of 83.8% and a ∂ statistic of 0.266.

We extracted the full 1,481 bp 16S rRNA gene from the 28M-7^T^ genome which had high similarity to several *Stappia* species (Table S2). Whole-genome-based phylogenetic analysis (TYGS) was used to determine 28M-7^T^’s closest relatives and calculate the dDDH. None of the related strains had a dDDH ≥ 70% or an ANI ≥ 95%, the thresholds above which two strains are considered the same species (Table S3). We generated a phylogenetic tree of the *Stappia* and *Hongsoonwoonella* type strains including 28M-7^T^, which revealed that this strain clusters with *Stappia indica,* a strain also isolated from the deep-sea (34) (Fig. 1). *Stappia sediminis* and *Stappia albiluteola* clustered separately with *Hongsoonwoonella zoysiae* and have been classified by the GTDB as members of the *Hongsoonwoonella* genus. The GTDB-Tk classified strain 28M-7^T^ (*Stappia* sp014252955) as a novel species, with its closest relative being *S. indica*. The environmental, diatom-associated strain *Stappia* sp. ARW1T was also identified as a representative of this same species (35, 36). Interestingly, the 28M-7^T^ assembly contained two contigs (168,820 bp and 69,494 bp) with high BLAST similarity to sequences from *Paracoccus binzhouensis* (37). These contigs were predicted to be plasmids by the sequence classifier tool PlasFlow v 1.1 (38). Both encoded complete MPF-type conjugation systems, along with key plasmid maintenance genes including *repA*, *parA*, and *copG*, confirming their conjugative nature, and suggesting one or more acquisition events. Phenotypic and physiological profiling data for 28M-7^T^ are shown in Table S4. An electron micrograph of a 28M-7^T^ cell is shown in Fig. S1.

#### Strain 28A-2^T^

**Fig 2:**
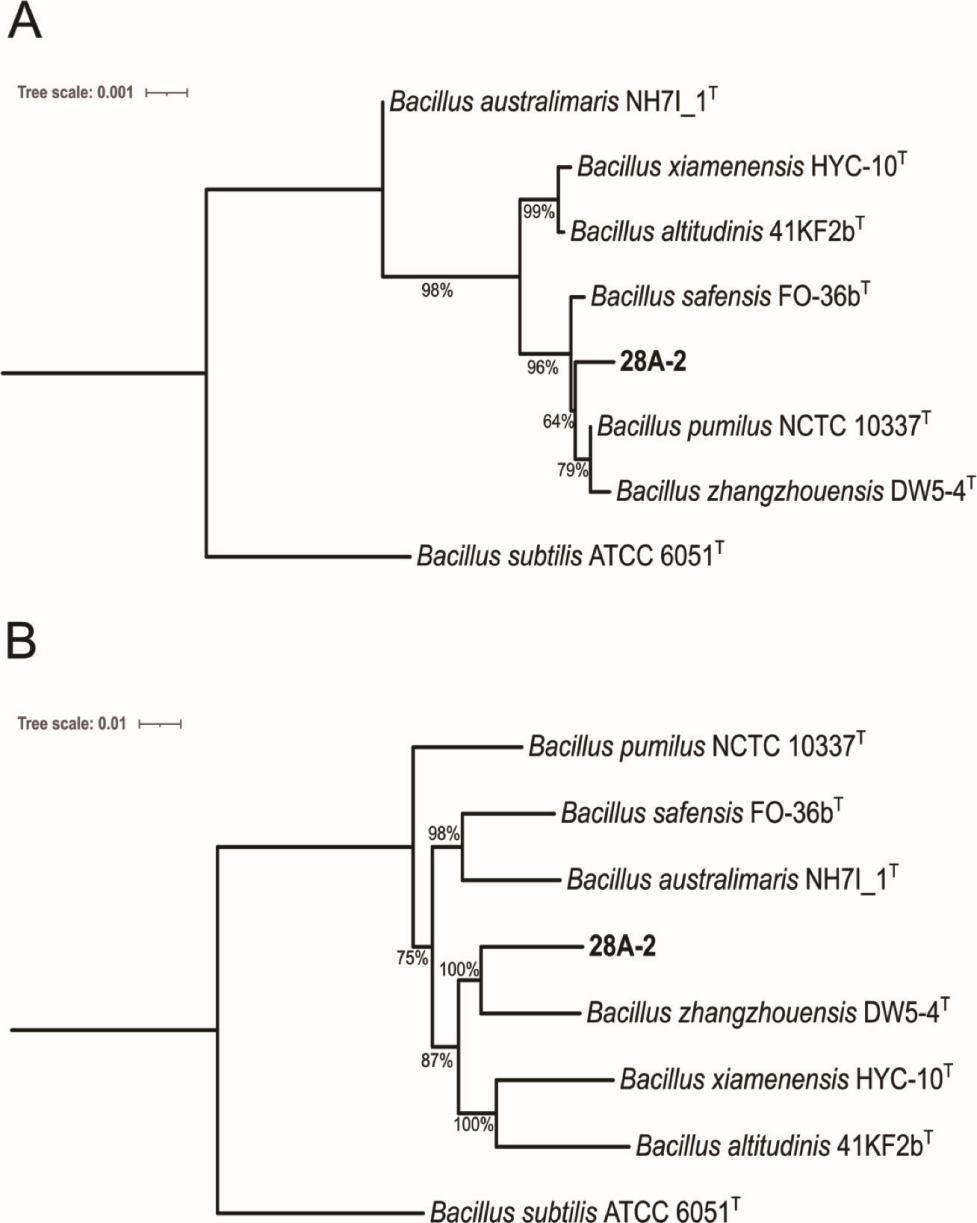
(**A**) 16S rRNA gene-based phylogenetic tree of strain 28A-2^T^. (**B**) Whole genome phylogenetic tree for strain 28A-2^T^, which we propose as *Bacillus crepusculi* sp. nov. Trees were constructed with TYGS (31) and prepared using iTOL (39). GBDP pseudo-bootstrap values from 100 replications are shown, values under 50% are not shown, branch lengths are scaled in terms of GBDP d5 distance formula. The tree was rooted using *Bacillus subtilis* ATCC 6051^T^ as the outgroup. The whole genome tree had an average branch support of 92% and a ∂ statistic of 0.264.

A complete 1,544 bp 16S gene was extracted from the 28A-2^T^ genome. It was found that its closest relatives were all members of the *Bacillus pumilus* group (Table S5). These species share 16S rRNA gene sequence identities of >99.5% (40). There have been several attempts to delineate the phylogenetics of this group based on DNA gyrase subunit B (*gyrB*) (41), concatenated housekeeping genes (40), and whole genome sequences (42). We produced phylogenetic trees for strain 28A-2^T^ and the other closely related members of the *B. pumilus* group based on 16S rRNA gene and whole genome similarity (Fig. 2). None of the closely related strains had a dDDH ≥ 70% or an ANI ≥ 95% indicating 28A-2^T^ represents a novel species within the *B. pumilus* group (Table S6). The GTDB-Tk classified strain 28A-2^T^ as a novel species (*Bacillus* sp014764715), with *Bacillus zhangzhouensis* its closest relative. Phenotypic and physiological profiling data for 28A-2^T^ are shown in Table S7. An electron micrograph of a 28A-2^T^ cell is shown in Fig. S2.

#### Strain 28M-43^T^

**Fig 3:**
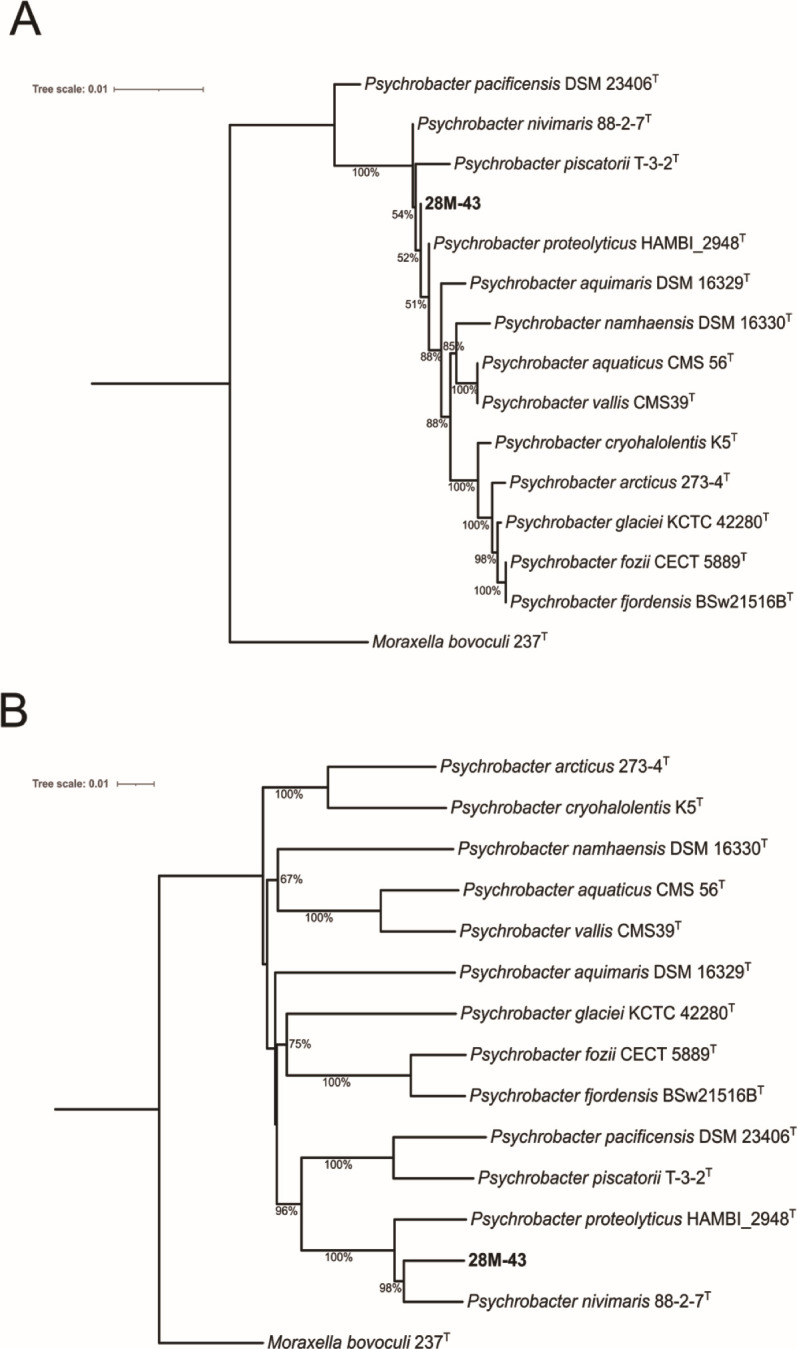
(**A**) 16S rRNA gene-based phylogenetic tree of strain 28M-43^T^. (**B**) Whole genome similarity tree for strain 28M-43^T^, which we propose as *Psychrobacter noctis* sp. nov. Trees were constructed with TYGS (31) and prepared using iTOL (39). GBDP pseudo-bootstrap values from 100 replications are shown, with branch lengths scaled in terms of GBDP d5 distance formula. The trees were rooted using *Moraxella bovoculi* 237^T^ as the outgroup. The phylogenomic tree had an average branch support of 80.5% and a ∂ statistic of 0.231.

For strain 28M-43^T^ a complete 1,534 bp 16S rRNA gene was successfully extracted from the genome. It shared 16S rRNA gene sequence identity of ≥98.7% with several *Psychrobacter* species (Table S8). We submitted the 28M-43^T^ genome assembly to the TYGS server to identify closely related strains and determine taxonomy. No related strains shared dDDH values ≥70% or ANI values ≥95% (Table S9). We generated a phylogenetic tree for 28M-43^T^ and the 13 closest *Psychrobacter* relatives of 28M-43^T^ (Fig. 3). The GTDB-Tk also classified strain 28M-43^T^ as a novel species (*Psychrobacter* sp014770435), with *Psychrobacter nivimaris* being its closest relative. Phenotypic and physiological profiling data for 28M-43^T^ are shown in Table S10. An electron micrograph of a 28M-43^T^ cell is shown in Fig. S3.

### Antibacterial activity screening of isolated strains

**Fig 4:**
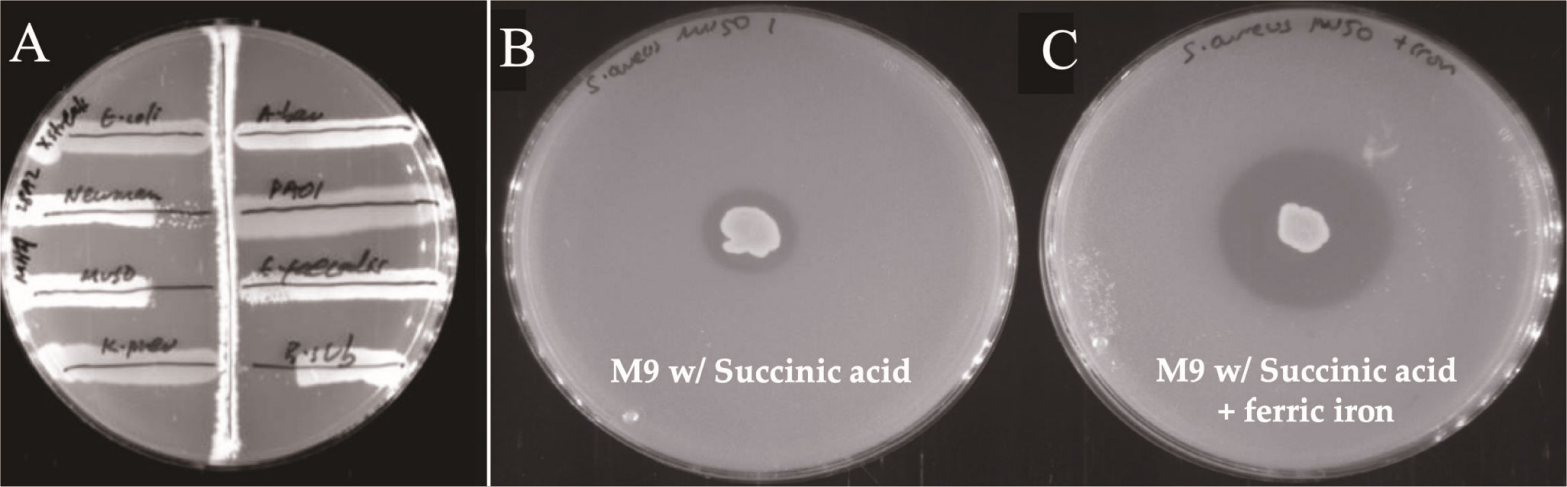
Antibacterial screening results. (**A**) Cross-streak assay on Mueller-Hinton agar showing the antibacterial activity of strain 28A-2^T^. Clockwise from the top left, *Escherichia coli* BW25113, *Acinetobacter baumannii* ATCC 19606, *Pseudomonas aeruginosa* PAO1, *Enterococcus faecalis* UB591, *Bacillus subtilis*, *Klebsiella pneumoniae* NCTC 5055, *Staphylococcus aureus* Mu50, and *Staphylococcus aureus* Newman are shown as streaks perpendicular to the central streak of strain 28A-2^T^. (**B**) Soft agar overlay for strain 28M-7^T^ grown on M9 agar with 10 g/L succinate for 7 days, overlaid with *S. aureus* Newman. (**C**) Soft agar overlay for strain 28M-7^T^ grown on M9 agar with 10 g/L succinate and ferric iron for 7 days, overlaid with *S. aureus* Newman.

To investigate the ability of 28M-7^T^, 28A-2^T^, and 28M-43^T^ to biosynthesize antimicrobial metabolites, antimicrobial activity screening was undertaken using a panel of clinically relevant bacterial pathogens. *Bacillus* 28A-2^T^ inhibited the growth of Gram-positive strains in these assays, including multidrug-resistant *Staphylococcus aureus* (Fig. 4A). Neither of the other two strains exhibited any antibacterial activity.

To explore the potential of activating silent BGCs, we designed a small OSMAC screen by changing the carbon source in the minimal M9 agar used for their cultivation (Table S11). *Stappia* 28M-7^T^ displayed antibiotic activity against *S. aureus* Newman only when grown with 10 g/L succinate (Fig. 4B). Interestingly, this activity was found to be further enhanced by the presence of ferric iron (Fig. 4C). *Psychrobacter* 28M-43^T^ did not display antibacterial activity under any condition tested and was, therefore, deprioritized for further study.

### Isolation of active compounds

#### Strain *Bacillus* 28A-2^T^

To investigate the antibiotic activity observed for *Bacillus* 28A-2^T^, we prepared organic extracts from bacteria grown on both agar plates and in liquid media. Agar plate extracts exhibited broad spectrum antimicrobial activity (Fig. S4) while broth extracts did not. We attempted activity-guided fractionation of the agar extracts using high-performance liquid chromatography (HPLC) but were unable to isolate the active constituent(s) in sufficient quantity to enable further characterization. We, therefore, adopted a bioinformatic approach using antiSMASH 7.0 to identify BGCs within the 28A-2^T^ genome. Eleven BGCs were identified, of which two possess significant sequence identity to known clusters associated with antimicrobial natural products, namely, the peptide antibiotic bacilysin (43) and a lichenysin-type biosurfactant (44). The anti-Gram-positive activity of strain 28A-2^T^ is likely attributable to bacilysin biosynthesis, as this compound has a more limited spectrum of activity than lichenysin, an observation in keeping with our antibacterial screening data (45, 46). That this activity was only observed in solid-state cultures is consistent with previous work showing that *Bacillus* species frequently redirect metabolic flux toward bioactive secondary metabolites, including lipopeptide biosurfactants, under surface-associated growth conditions (47).

#### Strain *Stappia* 28M-7^T^ 

**Fig 5:**
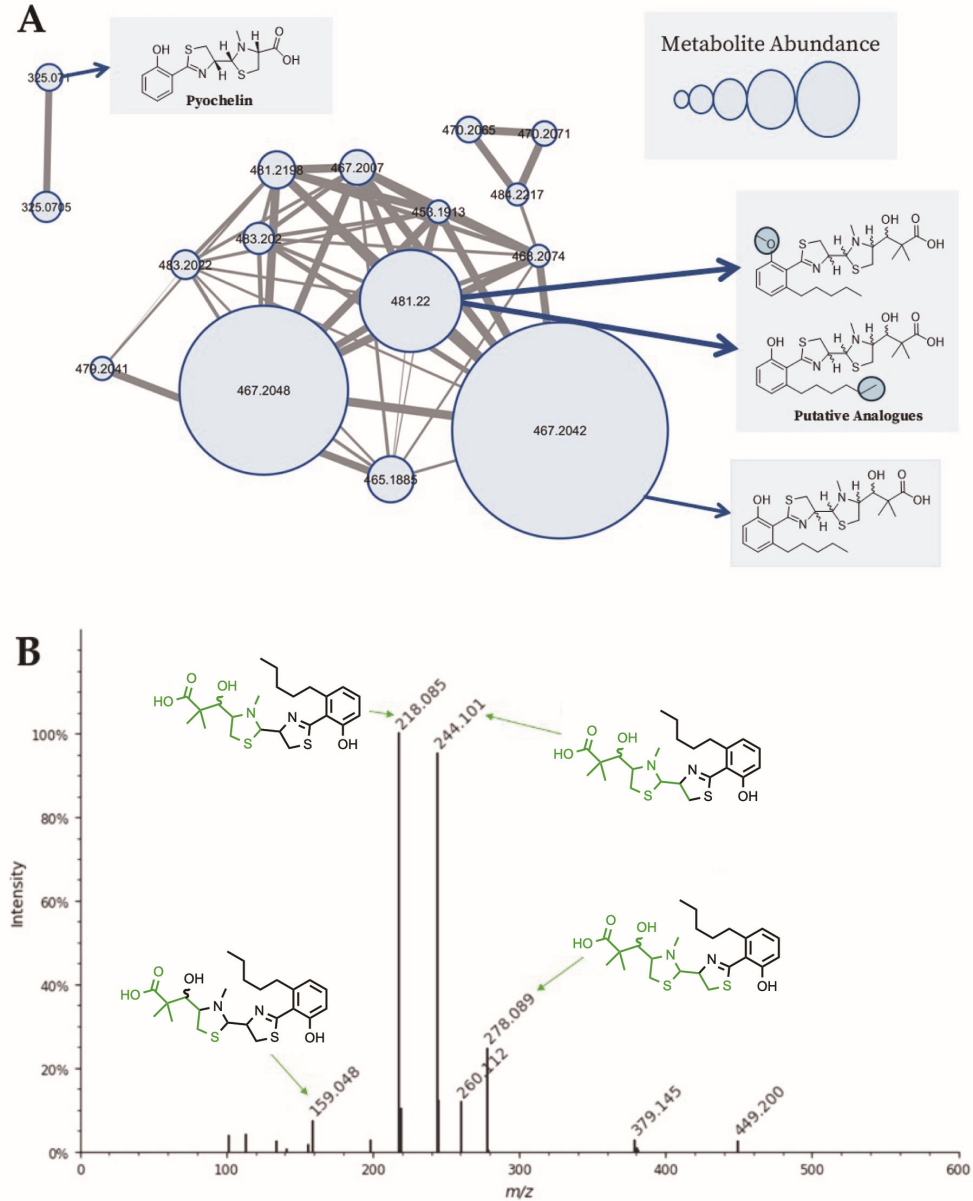
Feature-based molecular networking and *in silico* fragmentation analysis of the dominant metabolite from *Stappia* 28M-7 ^T^. (**A**) GNPS molecular network of selected MS/MS features from *Stappia* 28M-7^T^. The dominant metabolite isomer nodes *m/z* 467.20 [M+H]^+^ in a 14-node subnetwork believed to be agrochelin/massiliachelin. Related features at *m/z* 481.22 and 453.20 likely represent CH₂ chain length variants. Nodes are sized based on metabolite abundance based on the peak area. (**B**) MetFrag *in silico* fragmentation of agrochelin (NPA017357) against the experimental spectrum for *m/z* 467.205, with major fragments and assigned substructures shown in green.

To identify the natural product responsible for the antibiotic activity of *Stappia* 28M-7^T^, we first prepared a small-scale crude extract from cultures grown in M9 media supplemented with 10 g/L succinate. This extract was subjected to LC-MS/MS and the resulting data analyzed with feature-based molecular networking using the Global Natural Products Social Molecular Networking (GNPS) platform (48). The dominant metabolite *m/z* 467.205 [M+H]^+^ was present as two isomers, grouped into a network comprising 14 nodes (Fig. 5A). This *m/z* value corresponds to the reported molecular weight of the thiazole alkaloid siderophores massiliachelin (1) and agrochelin (2), previously isolated from *Massilia* and *Agrobacterium* spp., respectively (C₂₃H₃₄N₂O₄S₂; MW 466). Other metabolites within the network varied by ±14 Da, consistent with CH_2_ carbon chain length variations, with an abundant metabolite at 481.22 [M+H]^+^ (+14.0) and a less abundant metabolite of 453.2 [M+H]^+^ (−14.0), both directly linked to the central node. Furthermore, two low abundance features (*m/z* 325.0705) were identified in the extract with GNPS library matches to pyochelin (CCMSLIB00005724305; cosine score 0.87) although these did not cluster with the main network.

As no MS/MS reference spectra is available for massiliachelin or agrochelin, we used MetFrag (49) to compare the spectra with an *in silico* fragmentation prediction of agrochelin (NPA017357). This resulted in a final score of 1.0, indicating high agreement between predicted and observed fragments (Fig. 5B). Further analysis using Modifinder (50) identified the *m/z* 481.22 fragments matching those of *m/z* 467.205, with a +14 Da modification localized to the salicylic acid moiety, consistent with a previously unreported extended alkyl chain variant. Although Modifinder suggests a terminal +14 Da extension, odd-chain fatty acids have not been reported from the *Stappia* genus. An alternative is methyl esterification at the salicylate hydroxyl group. We did not detect a dimethyl ester, but neither can be definitively excluded.

#### Isolation and activity screening of the *Stappia* 28M-7^T^ metabolite

To enable compound isolation and to validate the source of the antibacterial activity of *Stappia* 28M-7 ^T^, we scaled up cultures (5 × 1 L) under identical growth conditions to those employed above. An organic extract was prepared from the culture supernatant and analyzed by LC-MS. Surprisingly, this revealed only a single dominant peak at *m/z* 467.205, indicative of altered metabolic flux within the bacterium during large scale fermentation. This change in metabolite profile could be a consequence of altered aeration, nutrient availability, or other factors. Purification by reverse-phase HPLC yielded sufficient quantity of the identified metabolite to enable NMR analysis and antibacterial testing.

We tested the purified compound for antibacterial activity and, in cases where activity was observed, determined the minimum inhibitory concentration (MIC; Table 2). The compound was tested both in isolation and following the addition of an equimolar quantity of ferric iron. The compound was found to exhibit antibacterial activity against *S. aureus* Newman and methicillin–vancomycin-resistant *S. aureus* Mu50 (51). Notably, this activity was enhanced fourfold when the compound was preincubated with iron, suggesting improved potency or increased cellular uptake in the iron bound form. Inhibition of growth was not observed against the Gram-negative species *E. coli*, *K. pneumoniae*, *A. baumannii*, and *P. aeruginosa*.

**TABLE 2:** Antibacterial activities of the purified *Stappia* 28M-7^T^ metabolite before and after incubation with equimolar ammonium iron(II) sulfate (Fe)^*a*^

Strain	Minimum inhibitory concentration (µg/mL)			
	Thiazole alkaloid		Ampicillin	Eq. Fe
	Alone	With eq. Fe		
*S. aureus* Newman	256	64	8	1,024
*S. aureus* Mu50	256	64	512	1,024

^*a*^ Ampicillin was used as a positive control and ammonium iron(II) sulfate as a negative control. MIC assays were performed in triplicate. 861 µg/mL of ammonium iron(II) sulfate is equimolar to 1,024 µg/mL of the isolated metabolite, the value given for “Eq. Fe”.

#### Structure elucidation of the *Stappia* 28M-7^T^ metabolite

**Fig 6:**
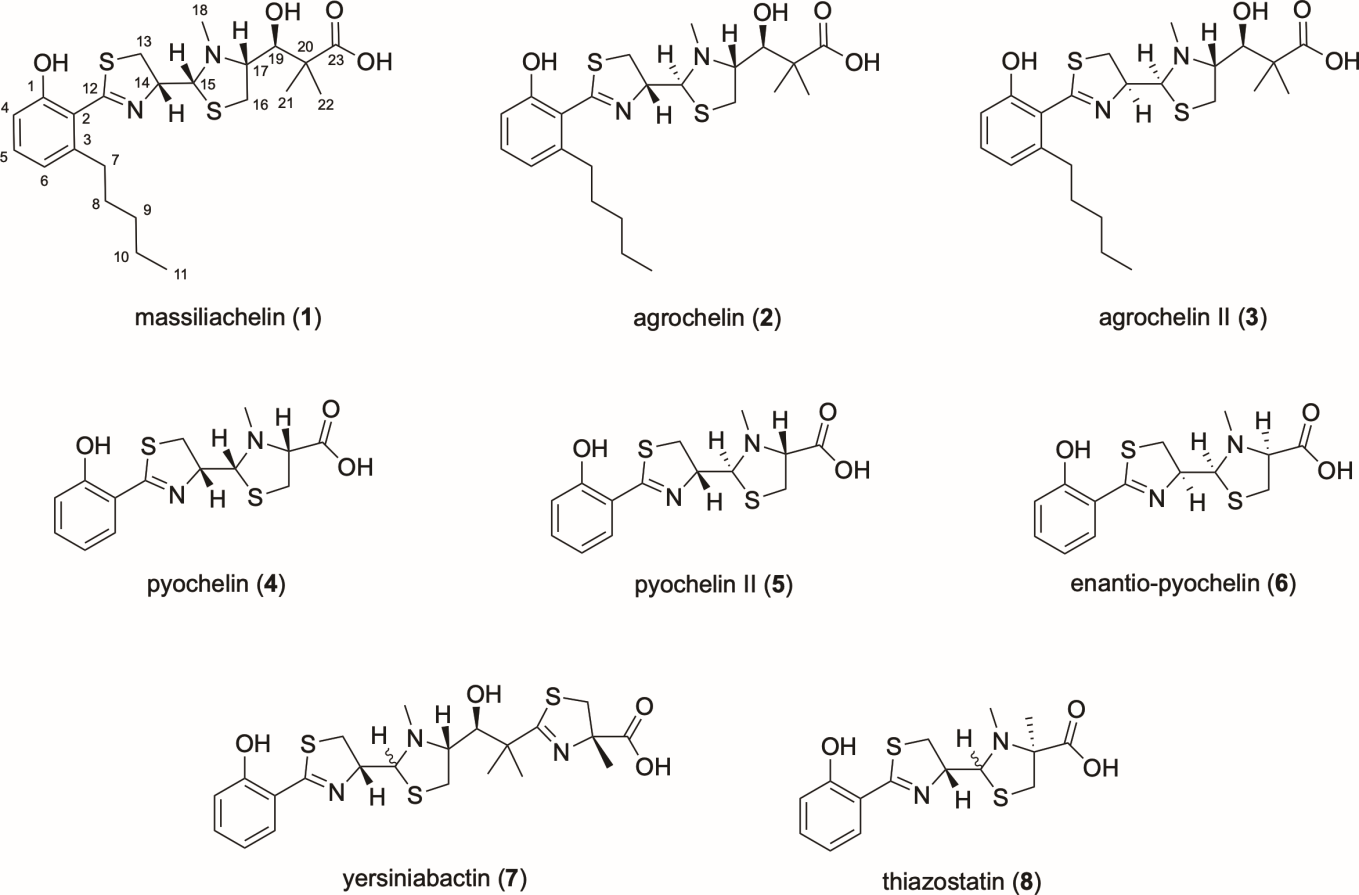
Comparative chemical structures of thiazole alkaloid natural products.

1H NMR analysis of the purified 28M-7^T^ metabolite indicated it to be closely related to the thiazole alkaloid siderophores massiliachelin (**1**) and agrochelin (**2**), in accord with our MS data (Fig. 6; Table S12). Indeed, full structural analysis by ^1^H, ^13^C, and 2D NMR revealed a compound with connectivity consistent with the 2D structures of these known metabolites (Tables S12 and S13). Importantly, however, our data were not entirely in keeping with those reported for either compound (52, 53) although they more closely resembled agrochelin (**2**). Additionally, the compound was found to be unstable, rapidly degrading into a mixture with poorly resolved ^1^H NMR signals. In an attempt to prevent degradation, we supplemented the product sample with ammonium iron (II) sulfate, in line with previous studies of the structurally related siderophores pyochelin I (**4**) and II (**5**), from *Pseudomonas aeruginosa* (Fig. 6) (54). Here, the equilibrium between pyochelin I (**4**) and pyochelin II (**5**) is driven toward pyochelin I (**4**) by the addition of divalent metal ions (55, 56), but no improvement in stability was observed here. The observed discrepancies between our data and those reported for massiliachelin/agrochelin could be due to subtle concentration or pH effects or may indicate that this compound is a novel diastereoisomer. We believed this compound may represent agrochelin II (**3**), with stereochemistry similar to that of enatio-pyochelin. This uncertainty prompted genome mining to identify the corresponding biosynthetic gene cluster.

### Genome mining of *Stappia* 28M-7^T^

In an effort to resolve the structure of the *Stappia* 28M-7^T^ metabolite, we employed an approach analogous to that used by Diettrich et al. (53), marrying bioinformatic predictions of metabolite chemistry with spectroscopic data. This approach required us to formally identify the BGC responsible for the biosynthesis of the natural product under investigation. The antiSMASH 7.0 platform identified nine BGCs in the 28M-7^T^ genome (Table 3), of which one (BGC 5) could be unambiguously assigned to the biosynthesis of a massiliachelin/agrochelin type thiazole alkaloid. The assignment was based on the distinctive domain architecture of this BGC as compared to that reported previously (53). The antiSMASH 7.0 pipeline also detected a *trans*-AT PKS/NRPS hybrid BGC (BGC 2) homologous to the *Stappia indica* sesbanimide C biosynthetic gene cluster, the only reported natural product from this genus (57). Notably, low-abundance features corresponding to sesbanimide A (*m/z* 328.1391 [M+H]^+^) and sesbanimide C (*m/z* 314.1598 [M+H]^+^) were both observed in our metabolomics data.

**TABLE 3:** BGCs identified in the genome of strain *Stappia* 28M-7^T^ by antiSMASH 7.0^*a*^

BGC	Type	Size (kb)	Predicted product
1	NRPS	41.7	Unknown
2	Thiopeptide, *trans*-AT PKS, T3PKS, NRPS	119	Sesbanimide R
3	RiPP-like	11.4	Unknown
4	T1PKS	46.6	Capsular lipooligosaccharide
5	NRPS, T1PKS	77.7	Agrochelin and massiliachelin
6	PKS-like, ectoine, NRPS-independent- siderophore	14.3	Ectoine and desferrioxamine relative
7	Terpene	20.9	Carotenoid
8	NAGGN	14.8	NAGGN
9	Phosphonate	41.6	Phosphinothricintripeptide

^*a*^ Assignment of predicted product is based on our own manual annotations as informed by the published literature. NRPS, nonribosomal peptide synthetase; *trans*-AT PKS, *trans*-acyltransferase polyketide synthase; T1/3PKS, type I/III polyketide synthase; RiPP-like, unspecified ribosomally synthesised and post-translationally modified peptide product; NAGGN, *N*-acetylglutaminylglutamine.

**Fig 7:**
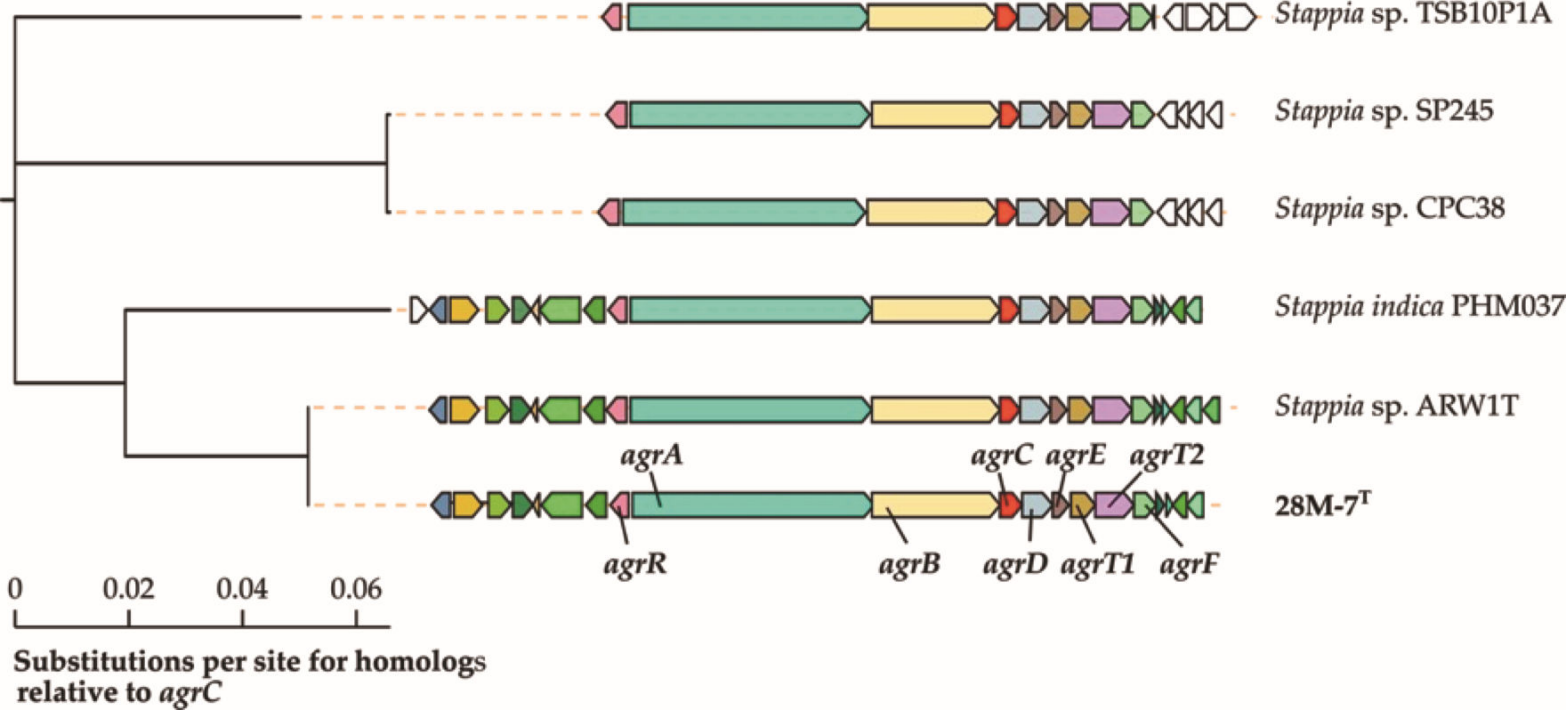
Cladogram for the minimal massiliachelin/agrochelin BGC from strain *Stappia* 28M-7^T^ compared to those of its closest relatives, generated using CORASON, with agrC as the query gene. Homologous genes are displayed using the same color. The annotated genes for the 28M-7^T^ massiliachelin/agrochelin BGC are shown in Table S13.

We used CORASON to identify massiliachelin/agrochelin BGCs in the 20 publicly available *Stappia* and *Hongsoonwoonella* genomes from NCBI and identified the cluster in five other *Stappia* strains (Fig. 7). By aligning the homologous genes in these BGCs, we were able to identify the nine genes conserved between all six BGCs, which we propose as the minimal massiliachelin/agrochelin BGC. This locus has been deposited in MiBIG (BGC0002764) (58). A full manual annotation of the massiliachelin/agrochelin BGC and surrounding genes in the strain 28M-7^T^ genome is shown in Table S14. To further explore the distribution of this cluster and briefly review the biosynthetic potential of the genus, we performed a gene cluster family (GCF) analysis using BiG-SCAPE2 (59). Seventeen assemblies were included, including four metagenome assembled genomes, restricted to GTDB-validated *Stappia* strains representing 10 species (Table S15). This analysis revealed the six strains containing the agrochelin BGC were split across four GTDB species clusters: “*Stappia quadratibracata”* sp. nov., *Stappia indica_A, Stappia sp900185725*, and *Stappia sp002722295* (Fig. S5). Interestingly, the massiliachelin BGC did not cluster with this GCF, likely due to a missing *agrB* homolog in the MiBIG accession (BGC0002687). In total, there were 181 BGCs across the 17 strains, consisting of 52 GCFs and 22 singletons. Notably, only two GCFs clustered with known BGCs in MiBIG, the sesbanimide BGC from *Stappia indica* PHM037 (57) and the ectoine BGC from the *Methylarcula marina* (60).

### BGC analysis and evidence for the biosynthesis of agrochelin II

With the *Stappia* 28M-7^T^ massiliachelin/agrochelin BGC sequence in hand, we next proceeded to assign the stereochemistry of the associated thiazole alkaloid using a bioinformatics-led approach. The presence of a methyltransferase-like epimerization (MT_e_) domain in the PKS-NRPS hybrid proteins responsible for the biosynthesis of thiazoline-containing siderophores is required for a D-configured ring (61). Conserved domain searching (62) revealed that the MT_e_ domain of AgrA from *Stappia* 28M-7^T^ is likely non-functional, due to the presence of an eight amino acid insertion within the active site region of this domain, consistent with a 14*S* configuration derived from incorporation of L-cysteine into the thiazoline ring. The presence of only a single adenylation domain for L-cysteine in AgrA further supports a 15*S* configuration equivalent to that of enantio-pyochelin (6) and thiazostatin (8) (Fig. 6) (63). The ketoreductase domain of AgrB has an aspartic acid at position 94 (Fig. S6), which is known, or predicted, to catalyze reduction resulting in a D-hydroxy (64); a proline at position 141 further supports this B-type stereochemistry (65). Intriguingly, the ketoreductase domains of HMWP1 (yersinibactin), MicC (micacocidin), and AgrA (*Stappia* 28M-7^T^ BGC) have a tyrosine to histidine substitution at residue 147. Tyrosine is part of the catalytic triad of ketoreductase domains required for activity, and these ketoreductase domains are predicted to be inactive (64–66). This observation conflicts with our spectroscopic data and suggests that ketoreduction at C19 is achieved via an alternative mechanism. Collectively, our spectroscopic and bioinformatics analyses are consistent with the assignment of the identified bioactive *Stappia* 28M-7^T^ metabolite as the previously unreported natural product agrochelin II (**3**) (Fig. 6).

## DISCUSSION

Here, we report the isolation and characterization of three bacterial species—“*Bacillus crepusculi”* sp. nov., “*Stappia quadratibracata”* sp. nov., and “*Psychrobacter noctis”* sp. nov.—from deep-sea sponges from the equatorial Atlantic Ocean. These findings form part of a wider collaborative study seeking to investigate the composition of the deep-sea sponge microbiome and its usefulness as a source of bioactive natural products (21). To explore the secondary metabolic potential of the three strains described herein antibacterial screening, in combination with OSMAC cultivation was undertaken, an experimental approach which proved effective in establishing the biosynthetic potential of the isolated bacteria. Both *Bacillus* 28A-2^T^ and *Stappia* 28M-7^T^ were found to biosynthesize metabolites with antibacterial activities. While the active metabolites from 28A-2^T^ could not be recovered in sufficient quantities to enable their formal identification, genome analysis identified BGCs encoding known antimicrobial compounds, findings consistent with our *in vitro* screening data (43, 44). In contrast, growth of *Stappia* 28M-7^T^ on succinate as a carbon source yielded a dominant metabolite, with spectroscopic and genomic data consistent with the previously unreported thiazole alkaloid agrochelin II, a close structural relative of the siderophores massiliachelin (1) and agrochelin (2). Intriguingly, complimentary untargeted metabolomics studies of *Stappia* 28M-7^T^ identified a number of additional distinct yet closely related analogs of agrochelin II, present within the fermentation media. The molecular masses of these compounds were consistent with both extended chain and truncated thiazole alkaloid analogs, along with the structurally related compound pyochelin. These findings contrast with those of Steinmetz et al., in their studies of *Massilia* sp. NR 4-1, where although multiple shunt products were identified, each retained the salicylate-carbon chain core, but were otherwise incomplete (67). The production of a broader portfolio of metabolites in *Stappia* 28M-7^T^ indicates a degree of promiscuity in the starter unit selection machinery of the associated biosynthetic pathway, consistent with previous mutasynthesis studies of both the yersiniabactin and pyochelin synthetases (68, 69). The biological relevance of relaxed starter unit selectivity in these pathways remains to be unambiguously established; however, such findings hint at the potential of these systems for use in the production of functionally optimized “non-natural” analogs.

Given the inherent chemical complexities common to natural product scaffolds, unambiguous structure elucidation remains a significant technical challenge. To address this issue, in our studies of *Stappia* 28M-7^T^, bioinformatic analysis has been successfully employed to resolve the structure of the dominant bioactive metabolite produced by this bacterium, agrochelin II. We propose that this approach be considered of broader utility in informing natural product structure elucidation, in particular in instances where compound instability and/or structural complexity precludes structure determination by spectroscopic means alone. The *Stappia* MTe domain harbors an eight-residue insertion near the predicted enzyme active site, which is not observed in the pyochelin, yersiniabactin, micacocidin, or massiliachelin BGCs. Disruption of MTe activity precluding L- to D-cysteine epimerization accounts for the 14*S* configuration observed in agrochelin II. While further investigation is needed to formally validate this hypothesis, it provides a plausible biosynthetic rationale for the stereochemical divergence of agrochelin II.

The genus *Stappia* remains a largely unexplored source of bioactive natural products, with studies to date having focused predominantly on the ecology, sulfur metabolism, and physiology of this bacterium (70, 71). As a consequence, the findings outlined herein constitute one of the few reported attempts to establish the usefulness of this bacterium as a source of novel secondary metabolites. Despite their widespread occurrence, comparatively little is known about the phylogenetic and physiological relationships amongst *Stappia* isolates, or how environmental factors influence their distribution and biological activities. The paucity of studies exploring the biosynthetic potential of *Stappia* is particularly surprising given that this genera resides within the *Rhodobacteraceae*/*Roseobacte*r clade, which contains a number of well-studied, biosynthetically gifted bacteria (72, 73). The absence of previous studies demonstrating “hit” identification in *Stappia* has undoubtedly disincentivized researchers to investigate natural product biosynthesis in this genera, further compounded by complexities associated with growth and cultivation, which are generally considered to be more challenging than for more historically favored producers (36). However, given the findings of this study, we suggest that *Stappia* ought to be reconsidered as an attractive target for future investigations seeking to identify clinically relevant bioactive metabolites.

Finally, our findings provide further support for the usefulness of deep-sea bioprospecting as an approach for the discovery of bioactive microbial natural products, including those with antimicrobial activities. This adds to a growing body of published literature, including from our own laboratories, which indicates that the distinctive environmental conditions experienced by deep-sea microbes translate into the acquisition of metabolic novelty (21–23). The value of integrating OSMAC screening into discovery platforms is also demonstrated, with succinate supplementation shown to be an effective strategy for the activation of silent BGCs, consistent with previous reports of succinate-induced siderophore production (74, 75). Collectively, these findings provide compelling evidence for the benefit of incorporating succinate into OSMAC screens as a general mechanism for BGC activation. Interestingly, iron-associated agrochelin II displayed enhanced antibacterial activity, potentially through increased cellular uptake or enhanced potency (76), which to our knowledge has not previously been reported for structurally related bioactive natural products. On the basis of this finding, we suggest value in revisiting previously reported thiazole alkaloids to establish if iron supplementation constitutes a general strategy of enhancing the antimicrobial activities of this family of compounds.

In summary, this study expands the known chemical space of *Stappia* natural products and marine siderophores, and demonstrates the value of integrated cultivation–screening-metabolomics–genomics pipelines for the discovery of bioactive secondary metabolites.

### Descriptions of new species

#### Description of *Stappia quadratibracata* sp. nov.

*Stappia quadratibracata*: qua.dra.ti.bra.ca’ ta. L. fem. nom. n. *quadrata*, square or squared, L. fem. adj. *bracata*, wearing trousers or breeched, N.L. fem. adj. *quadratibracata*, square-trousered, for the character SpongeBob SquarePants, referencing the sponge from which the type strain was isolated.

Cells are Gram-stain negative, aerobic, flagellated rods approximately 0.9–1.3 × 0.3–0.5 µm in size. On marine agar 2216, colonies are pinkish-beige, shiny, viscous, opaque, round, and convex with entire margins after 72 h growth at 28 °C. Growth occurs on unbuffered nutrient agar at 15–37°C, at pH 5–12, and with 0%–8% (wt/vol) NaCl. Using the API 20NE system, cells are positive for oxidase, nitrate respiration, aesculin hydrolysis, gelatinase, and β-galactosidase activities, and negative for denitrification, indole production, fermentation, arginine dihydrolase, and urease activities. With the API 20NE system, positive for nitrate respiration, denitrification, and oxidase activities; negative for indole production, fermentation, arginine dihydrolase, urease, esculin hydrolysis, gelatinase, and β-galactosidase activities. D-Glucose, D-mannitol, adipic acid, malic acid, citric acid, and phenylacetic acid are assimilated as sole carbon sources; L-arabinose, D-mannose, *N*-acetylglucosamine, D-maltose, gluconic acid, and capric acid are not.

The type strain is 28M-7^T^ (= NCIMB 15403^T^ = DSM 112057^T^), which was isolated from a deep-sea sponge from the Knipovich seamount in the Atlantic Ocean. The genome size of the type strain is 5.13 Mb, and its genomic DNA G + C content is 66.7 mol %.

#### Description of *Bacillus crepusculi* sp. nov.

*Bacillus crepusculi*: cre’ pus.cul.i. L. gen. n. *crepusculi* of the twilight, referring to the “twilight zone” of the ocean from which the type strain was isolated.

Cells are Gram-stain positive, aerobic, spore-forming rods approximately 2.7–3.5 × 0.6–1.0 µm in size and motile by means of peritrichous flagella. Exhibits sliding surface motility on media with 1% agar or less. Inhibits the growth of *S. aureus*, *E. faecalis*, and *B. subtilis* after growth on Mueller-Hinton or actinomycetes isolation agar for 3 days. On Luria-Bertani agar (LBA), colonies are opaque, off-white, shiny, circular, and flat with undulate margins after 48 h growth at 28 °C. Growth occurs on unbuffered LBA at 15–45°C, at pH 5–12, and with 0%–10% (wt/vol) NaCl. Using the API 20NE system, cells are positive for oxidase, nitrate respiration, aesculin hydrolysis, gelatinase, and β-galactosidase activities, and negative for denitrification, indole production, fermentation, arginine dihydrolase, and urease activities. With the API 20NE system, D-Glucose, L-arabinose, D-mannose, D-mannitol, *N*-acetylglucosamine, D-maltose, gluconate, malate, and citrate are assimilated as sole carbon sources, but capric acid, adipic acid, and phenylacetate are not.

The type strain is 28A-2^T^ (= NCIMB 15401^T^ = DSM 111873^T^), which was isolated from a deep-sea sponge from the Vayda seamount in the Atlantic Ocean. The genome size of the type strain is 3.70 Mb, and its genomic DNA G + C content is 41.7 mol %.

#### Description of *Psychrobacter noctis* sp. nov.

*Psychrobacter noctis*: noc’ tis. L. gen. n. *noctis* of the night, referring to the “midnight zone” of the ocean from which the type strain was isolated.

Cells are Gram-stain negative, aerobic, non-spore-forming cocci approximately 1.2–1.6 µm in diameter arranged in chains. On nutrient agar, colonies are opaque, cream, shiny, circular, and convex with entire margins after 72 h growth at 28°C. Growth occurs on unbuffered LBA at 4–34°C, at pH 5–12, and with 0%–12% (wt/vol) NaCl. Using the API 20NE system, cells are positive for oxidase activity and negative for nitrate respiration, denitrification, indole production, fermentation, arginine dihydrolase, urease, aesculin hydrolysis, gelatinase, and β-galactosidase activities. With the API 20NE system, D-Glucose, L-arabinose, D-mannose, D-mannitol, *N*-acetylglucosamine, D-maltose, gluconate, capric acid, adipic acid, malate, citrate, and phenylacetate are not used as sole carbon sources.

The type strain is 28M-43^T^ (= NCIMB 15404^T^ = DSM 112058^T^), which was isolated from a deep-sea sponge from the Vayda seamount in the Atlantic Ocean. The genome size of the type strain is 3.12 Mb and its genomic DNA G + C content is 43.0 mol %.

## MATERIALS AND METHODS

### Isolation and antimicrobial screening of bacteria

The sponges, Demosponge B0078 (971 m, Knipovich Seamount), Hexactinellid B01641 (868 m, Vayda Seamount), and Demosponge B01642 (1,483 m, Vayda Seamount) were processed, and bacteria were isolated from them as previously described (21). In short, we rinsed the sponges thrice with artificial seawater to remove loosely associated bacteria and then ground each with artificial seawater in a sterile pestle and mortar. We made serial dilutions of the resulting solutions, spread the dilutions on a range of agar-based media, and incubated them at 4°C and 28°C for 2–10 weeks. We selected individual colonies and re-streaked them to yield axenic cultures, which we used to inoculate Microorganism Preservation System Protect Cryotubes (Technical Service Consultants Ltd, Heylood, UK) for storage at −70°C. Strains 28M-7^T^ and 28M-43^T^ were isolated on marine agar 2216 (BD Difco) at 28°C, and 28A-2^T^ was isolated on actinomycetes isolation agar (Sigma-Aldrich) at 28°C.

The bacteria isolated from the sponges were screened for antibacterial activity with a soft agar overlay assay, as previously described (21). Initially, we grew the sponge bacteria on agar under the same conditions that they were isolated under and overlaid the cultures with suspensions of bacteria pathogens suspended in soft agar. We then selected a subset of our sponge bacteria, including strains 28M-7^T^, 28A-2^T^, and 28M-43^T^ for an OSMAC screen, as described in Williams et al. (21). Briefly, we grew 90 strains on M9 agar (Serva Electrophoresis GmbH, Heidelberg, Germany) with a range of different carbon sources (colloidal chitin, dextrose, glycerol, mannitol, soluble starch, and succinate) for a week and then overlaid them with pathogens in soft agar. The sponge isolates which fell within a zone of inhibition were grown as monocultures on the relevant agar and screened again in triplicate—those which continued to produce zones of inhibition were considered active. A summary of the OSMAC screening results for 28M-7 ^T^ is provided in Table S11, and the compositions of the media used in this study are shown in Table S16.

### Genome sequencing and assembly

For genome sequencing, each strain was cultured in 10 mL of broth culture in a shaking incubator for three days at 28°C and 180 rpm. The liquid broth used for this step was equivalent to the agar composition used for the original isolation. Cells were harvested by centrifugation and genomic DNA extracted using a GenElute Bacterial Genomic DNA Kit (Sigma-Aldrich, St. Louis, MO, USA). The concentration and purity of the genomic DNA was assessed spectroscopically by measuring the absorbance at 230, 260, and 280 nm, and gel electrophoresis on a 0.8% agarose gel was used to assess the degree of DNA shearing.

Sequencing was performed by the Genomics Facility at the University of Bristol. One microgram of DNA was used to generate a paired end TruSeq library. This was indexed with a Nextera XT Index Kit prior to shotgun sequencing on an Illumina MiSeq platform (Illumina, San Diego, USA). Illumina reads were trimmed with Trim Galore v 0.4.4 (77) using a PHRED score of 20. In parallel, 1 µg of the same genomic DNA sample was also sequenced with a R9 MinION flow-cell indexed with the EXP-NBD103 kit (Oxford Nanopore Technologies, Oxford, UK) following the manufacturer’s instructions. Nanopore reads were basecalled with Albacore v 1.2.5 with adaptor trimming and demultiplexing via Porechop (Porechop, RRID: SCR_016967). Hybrid genome assemblies were generated with the Unicycler pipeline v 0.4.6 using default parameters in “normal” mode, first performing a de Bruijn graph assembly using the Illumina paired-end reads with SPAdes (78) and then using a semi-global aligner to align long Nanopore reads into a contiguous single assembly graph (79). Assemblies were quality checked with CheckM v 1.1.6 (80) and BUSCO v 5.3.2 (81). PlasFlow v 1.1.0 (38) was used to check for likely plasmids in assemblies via the web platform (https://usegalaxy.eu/) (82). Mobilome-related genes, including conjugation systems and plasmid maintenance genes, were annotated using Prokka v1.13 (83).

### Taxonomy

Genome assemblies were annotated with Prokka v 1.13 (83). Nucleotide sequences for complete 16S rRNA genes were used for nucleotide BLAST searches of the NCBI 16S rRNA database. The Genome Taxonomy Database Toolkit (GTDB-Tk v 2.4.1) was used to assign taxonomy by classifying the assemblies in the GTDB reference tree v. R220 (32, 33). For taxonomic placement with recognized type strains, we used the Type Strain Genome Server v 401 (TYGS) (31) pipeline. TYGS implements the Genome BLAST Distance Phylogeny (GBDP) method to infer whole genome phylogenetic trees (29). Intergenomic distances were calculated with the GBDP d5 distance formula, and trees inferred with FastME 2.1.4 with subtree pruning and regrafting postprocessing (84, 85). Phylogenetic tree branch support was assessed using 100 GBDP pseudo-bootstrap replicates, and robustness of treelikeness was assessed using ∂ statistic, where low δ values indicate strong treelikeness and high branch support (86). Only validly published type strains were included in phylogenetic analyses, consistent with standard practice for novel species descriptions. Closest reference strains were selected based on TYGS and GTDB-Tk results. Trees were rooted using an appropriate outgroup and visualized using the Interactive Tree of Life v 5.7 (iTOL) (39). ANI values were calculated using fastANI v 1.31 (30).

The API 20NE kit (BioMérieux, Marcy-l'Étoile, France) was used to assess enzymatic activities and carbon source utilization for isolated strains, following the manufacturer’s instructions and repeating the assays in triplicate. The pH of the standard agars was adjusted to 5, 6, 7, 8, 9, 10, 11, and 12. The NaCl content of the standard agars was adjusted to 0%, 2%, 4%, 6%, 8%, 10%, and 12% (wt/vol). Standard agar plates were incubated at 4°C, 15°C, 20°C, 28°C, 37°C, 42°C, and 45°C and incubated for 3 days (or a month for 4°C). All culturing was performed in triplicate.

For transmission electron microscopy (TEM), 5 µL of the liquid culture after 72 h growth was drop cast on to a carbon-coated formvar TEM grid pre-dipped in 2.5% glutaraldehyde. After 5 min, excess material was wicked away and grids washed twice in distilled water before imaging with an FEI Tecnai12 BioTwin TEM with an FE CETA camera.

The three strains described in this study are publicly available from the NCIMB (UK) and DSMZ (Germany) culture collections under the following accessions: *Stappia quadratibracata* 28M-7ᵀ (= NCIMB 15403ᵀ = DSM 112057ᵀ), *Bacillus crepusculi* 28A-2ᵀ (= NCIMB 15401ᵀ = DSM 111873ᵀ), and *Psychrobacter noctis* 28M-43ᵀ (= NCIMB 15404ᵀ = DSM 112058ᵀ)

### Metabolomics

For metabolomics analysis, a primary culture of *Stappia* 28M-7^T^ was grown in M9 media plus 10 g/L succinate (Sigma-Aldrich) (pH 7.0) for 4 days at 28°C at 180 rpm. Five milliliters of this primary was inoculated into 100 mL of the same media in a 250 mL Erlenmeyer flask and incubated for a further 8 days under the same conditions. Cells were harvested by centrifugation (4,000 × *g*, 20 min, 4°C), and the remaining supernatant was mixed with an equal volume of ethyl acetate (Sigma-Aldrich). The aqueous phase was discarded and anhydrous MgSO_4_ (Sigma-Aldrich) was added to the organic phase to bind any remaining water. The organic phase was filtered and concentrated *in vacuo* until dry, before redissolving the remaining extract in 1 mL LC–MS grade methanol (Sigma-Aldrich). LC-MS/MS data were acquired in electrospray ionization (ESI) positive mode on a Waters XEVO G2-XS QTof (Waters, Milford, MA, USA) with a C18 reverse-phase column (Phenomenex, 150 × 15 mm, 5 µm; Phenomenex, Torrance, CA, USA). A linear gradient of 5% MeCN to 95% MeCN with 0.1% formic acid over 20 min at a flow rate of 0.25 mL/min (Sigma-Aldrich). Waters .raw data files were converted to mzML format using MSConvert (v 3.0), and data were processed in MZmine (mzmine.io v 4.7.8) (87) using the mzmine wizard with default parameters for the instrument and applying a strict MS¹ intensity cut-off of 1 × 10⁵. Feature-based molecular networking was performed on GNPS2 (Workflow: 2025.07.09) (88). The molecular network was visualized using Cytoscape (v 3.10) (89). MetFrag (v 2.6.10) was used to generate *in silico* fragmentation via https://msbi.ipb-halle.de/MetFrag/ (49), using the agrochelin structure from NPAtlas (NPA017357) (90). Finally to identify modifications within the network, Modifinder (50) was run on the GNPS2 platform using the agrochelin SMILES to identify chemical modifications of related nodes.

### Natural product isolation

For culture scale-up, a primary 300 mL culture of 28M-7^T^ was grown in M9 media plus 10g/L succinate adjusted to pH 7.0 for 4 days at 28°C on a shaking table at 180 rpm. Fifty milliliters of this primary culture was inoculated into each of 5 × 1 L culture broth in 2.5 L glass flasks and grown on for a further 8 days. Cells were harvested by centrifugation, and the supernatant was mixed with an equal volume of ethyl acetate (Sigma-Aldrich). The aqueous phase was discarded, and organic phase was dried with anhydrous MgSO_4_, filtered, and concentrated *in vacuo* redissolved in 2 mL methanol (Sigma-Aldrich). LC-MS data were obtained on a Waters LCMS system comprising Waters 2767 autosampler, Waters 2545 HPLC pump, Waters 2998 Diode Array detector, Waters 2424 ELS detector, and Waters Quatro Micro mass spectrometer. HPLC grade H_2_O and MeCN were added with 0.05% formic acid as the solvent system. Analytical LC-MS data were obtained using a Phenomenex Luna column (C18, 250 × 4.60 mm, 5 μm) at a flow rate of 1 mL/min, with a gradient of 5% MeCN to 95% MeCN over 20 min. Preparative HPLC purification was carried out using a Phenomenex Kinetex column (C18, 250 × 21.20 mm, 5 μm) at a flow rate of 16 mL/min, with a gradient of 30% to 40% of MeCN over 20 min. HR-ESI-MS data were obtained on a Bruker Daltonics micrOTOF II instrument. NMR data were collected on a Bruker Cryo500 NMR spectrometer.

### Natural product antibacterial assays

To determine the minimal inhibitory concentrations (MIC) of agrochelin II, we followed the broth dilution protocol outlined by the European Committee for Antimicrobial Susceptibility Testing (91). We performed the assay in triplicate, using ampicillin as a positive control, and ammonium iron(II) sulfate and methanol as negative controls. We tested purified agrochelin II alone and preincubated with equimolar ammonium iron(II) sulfate. We determined MIC values for a panel of clinically relevant bacteria: *Staphylococcus aureus* Newman and Mu50, *Pseudomonas aeruginosa* PA01, *Klebsiella pneumoniae* NCTC 5055, *Acinetobacter baumannii* ATCC 19606, and *Escherichia coli* BW55113.

### Genome mining

Gene cluster analysis of the isolated strains used antiSMASH v.7.0 (92) which utilizes MiBIG 3.0 (93). CORASON (94) was used to define the core agrochelin BGC which was further annotated and defined based on gene conservation within *Stappia*. For the comparative gene cluster family analysis of the *Stappia* genus, only genomes which were assigned to the GTDB *Stappia* genus were included (Table S15). Genomes were annotated with antiSMASH (95) (v.8.0.4) and clustered into gene cluster families with BiG-SCAPE v.2.0.0 run in mixed mode (59) and with MiBIG 4.0 reference BGCs (58). The GCF cluster cut-off value was set to 0.35, and the cluster network was exported and visualized in Cytoscape (v 3.10) (89). Amino acid sequences of the MTe domain were extracted and aligned by MAFFT –auto (v 7.490) (96).

## Data Availability

Genome and 16S rRNA gene GenBank accession numbers for the reported strains are as follows: *Stappia quadratibracata* 28M-7^T^ genome: JACMIA000000000.1; assembly: GCA_014252955.1; 16S rRNA gene: MT999484.1: *Bacillus crepusculi* 28A-2‑^T^ genome: JACXXE000000000.1; assembly: GCA_014764715.1; 16S rRNA gene: MW003702.1: *Psychrobacter noctis* 28M-43^T^ genome: CP061739.1, assembly: GCA_014770435.1; 16S rRNA gene: MW003704.1. The mass spectrometry data supporting this study have been deposited in the MassIVE repository under accession number MSV000098784. The MS2 spectra of agrochelin was deposited in the GNPS library under the accession CCMSLIB00016341469.

## SUPPLEMENTAL MATERIAL

Supplemental material aem.01877-25-s0001.pdf Tables S1 to S16, Fig. S1 to S6, and compound spectra.

ASM does not own the copyrights to Supplemental Material that may be linked to, or accessed through, an article. The authors have granted ASM a non-exclusive, world-wide license to publish the Supplemental Material files. Please contact the corresponding author directly for reuse.
