# Supplementary material for: OSMAC guided bioprospecting of Atlantic sponges reveals novel bacterial species and evidence for the antimicrobial thiazole alkaloid agrochelin II
Source: Appl Environ Microbiol. 2026 Feb 17;92(3):e01877-25. doi: 10.1128/aem.01877-25 (PMC12997852; doi:10.1128/aem.01877-25)
Supplement: Supplemental material — Tables S1 to S16, Fig. S1 to S6, and compound spectra. [file aem.01877-25-s0001.pdf]

Supplementary Materials

OSMAC guided bioprospecting of Atlantic sponges reveals novel bacterial species and evidence for the antimicrobial thiazole alkaloid agrochelin II

Sam E. Williams, Henry L. Stennett, Andrew J. Devine, Zhongshu Song, Catherine R. Back, Luoyi Wang, Judith Mantell, Chris Neal, Mark A. Jepson, Angela E. Essex-Lopresti, Jonathan D. Sellars, James E. M. Stach, Christine L. Willis, Paul Curnow, and Paul R. Race

Table S1. Genome assembly quality assessment using CheckM and BUSCO. BUSCO checked against the order Rhizobiales (28M-7^T), the order Bacillales (28A-2^T) and the order Pseudomonadales (28M-43^T).

Strain	NCBI RefSeq Accession	CheckM Completeness	CheckM Contamination	BUSCO %
**28M-7^T**	NZ_JACMIA000000000.1	99.89%	0.47%	99.3%
**28A-2^T**	NZ_JACXXE000000000.1	99.59%	0.26%	99.8%
**28M-43^T**	NZ_CP061739.1	99.95%	0.92%	99.5%

Table S2. The species most closely related to strain 28M-7^T based on 16S rRNA gene sequences.

BLAST relative	Identity	Query coverage*	E value
Stappia indica B106	99.93 %	97 %	0.0
Stappia stellulata NBRC 15764	98.51 %	95 %	0.0
Stappia taiwanensis CC-SPIO-10	98.22 %	91 %	0.0
Stappia stellulata IAM 12621	97.88 %	95 %	0.0
Stappia albiluteola F7233	96.88%	95%	0.0

* All partial sequences, hence, coverage < 100 %.

Table S3. Phylogenomics of strain 28M-7^T based on digital DNA-DNA hybridization (dDDH) and average nucleotide identity (ANI). Strains with dDDH values below 70 % and ANI values below 95 % can be considered as separate species to strain 28M-7^T.

Strain	dDDH (%)	ANI (%)	% G+C difference
Stappia indica SBBC 49	48.5	93.6	0.39
Stappia taiwanensis CCM7757	21.1	80.9	1.26
Stappia stellulata DSM 5886	20.9	80.8	1.92
'Stappia' albiluteola F7233	20.7	79.6	3.35
Paracoccus binzhouensis wg1T	23.7	78.6	1.05
'Stappia' sediminis BGMRC 2046	19.5	78.5	5.84
Hongsoonwoonella zoysiae SY4-7	19.5	78.3	6.28

Table S4. A comparison of strain 28M-7^T and the four extant *Stappia* species based on phenotypic and physiological characterisation. Strains: 1, *Stappia indica* B106^T; 2, *Stappia stellulata* IAM 12621^T; 3, *Stappia taiwanensis* CC-SPIO-10^T; 4, *Stappia albiluteola* F7233^T. The data for *S. indica*, *S. stellulata*, *S. taiwanensis*, and *S. albiluteola* were taken from the literature. Growth conditions were tested on unbuffered nutrient agar. API 20NE strips were used to determine the enzymatic activities of 28M-7^T and its ability to grow with different substrates as sole carbon sources. A positive result is indicated by '+', a negative result by '-', a weakly positive result by 'w', and no data by 'ND'.

Enzymatic activities	28M-7^T	1	2	3	4
Reduction of nitrates to nitrites	+	+	-	-	+

Reduction of nitrates to dinitrogen	+	-	+	-	ND
Indole production	-	-	-	-	+
Fermentation	-	-	w	-	ND
Arginine dihydrolase	-	-	-	-	-
Urease	-	-	-	-	+
Aesculin hydrolysis	-	-	-	-	+
Gelatinase	-	-	-	-	-
β-Galactosidase	-	-	-	+	-
Oxidase	+	+	+	+	+
**Growth substrates**	**28M-7^{T}**	**1**	**2**	**3**	**4**
D-Glucose	+	+	+	+	+
L-Arabinose	-	-	+	+	+
D-Mannose	-	-	+	-	+
D-Mannitol	+	+	+	-	ND
N-Acetylglucosamine	-	-	ND	+	ND
D-Maltose	-	-	+	-	+
Gluconic acid	-	+	+	-	ND
Capric acid	-	ND	ND	ND	ND
Adipic acid	w	+	ND	-	ND
Malic acid	+	ND	ND	+	ND
Citric acid	+	+	+	+	ND
Phenylacetic acid	w	ND	ND	-	ND
**Growth conditions**	**28M-7^{T}**	**1**	**2**	**3**	**4**
Temperature range	15–37 °C	4–42 °C	5–37 °C	10–45 °C	15–40 °C
pH range (unbuffered)	5–12	ND	ND	6.5–11.5	5.5–8.5
Salinity (w/v NaCl)	0–8 %	0.5–11 %	ND	1–6 %	0–9 %
**Morphology**	**28M-7^{T}**	**1**	**2**	**3**	**4**
Cell shape	Rods	Rods	Rods	Rods	Rods
Cell dimensions	0.3–0.5 x 0.9–1.3 µm	1 x 1 µm	0.6–1.0 x 2.0–4.0 µm	0.5 x 0.8–1.0 µm	0.3–0.4 x 1.2–1.4 µm
Flagellated?	Yes	Yes	Yes	Yes	Yes
Colony colour	Pink-beige	Grey-white	Beige-brown	Beige	Beige

Table S5. The B*acillus* species most closely related to strain 28A-2^T based on 16S rRNA gene sequences.

BLAST relative	Identity	Query coverage*	E value
Bacillus pumilus NBRC 12092	99.80 %	95 %	0.0
Bacillus safensis NBRC 100820	99.80 %	95 %	0.0
Bacillus zhangzhouensis MCCC 1A08372	99.74 %	97 %	0.0
Bacillus australimaris MCCC 1A05787	99.67 %	97 %	0.0
Bacillus altitudinis 41KF2b	99.53 %	95 %	0.0

* All partial sequences, hence, coverage < 100 %.

Table S6. Phylogenomics of strain 28A-2^T based on digital DNA-DNA hybridization (dDDH) and average nucleotide identity (ANI). Strains with dDDH values below 70 % and ANI values below 95 % can be considered as separate species to strain 28A-2^T. The remaining strains had dDDH values below 44.2 %.

Strain	dDDH (%)	ANI (%)	% G+C difference
Bacillus pumilus 51_5il	51.2	93.08	0.06
Bacillus zhangzhouensis DW5-4	50.4	92.84	0.31
Bacillus pumilus 7P	44.3	91.49	0.19
Bacillus pumilus ZB201701	44.2	91.49	0.16
Bacillus pumilus 3-19	44.2	91.49	0.18

Table S7. A comparison of strain 28A-2^T and its closest relative based on phenotypic and physiological characterisation. The data for *B. zhangzhouensis* DW5-4^T (strain 1) were taken from the literature. Growth conditions were tested on unbuffered Luria-Bertani agar. API 20NE strips were used to determine the enzymatic activities of 28A-2^T and its ability to grow with different substrates as sole carbon sources. A positive result is indicated by '+', a negative result by '-', a weakly positive result by 'w', and no data by 'ND'.

**Enzymatic activities**	**28A-2^T**	**1**
Reduction of nitrates to nitrites	+	-
Reduction of nitrates to dinitrogen	-	-
Indole production	-	-
Fermentation	-	+
Arginine dihydrolase	-	+
Urease	-	+
Aesculin hydrolysis	+	+
Gelatinase	+	+
β-Galactosidase	+	+
Oxidase	+	+
**Growth substrates**	**28A-2^T**	**1**
D-Glucose	+	+
L-Arabinose	+	+
D-Mannose	+	+
D-Mannitol	+	+
N-Acetylglucosamine	+	+
D-Maltose	+	-
Gluconic acid	+	+
Capric acid	-	-
Adipic acid	-	-

Malic acid	+	+
Citric acid	+	+
Phenylacetic acid	-	-
**Growth conditions**	**28A-2^T**	**1**
Temperature range	15–45 °C	8–45 °C
pH range (unbuffered)	5–12	5–11
Salinity (w/v NaCl)	0–10 %	0–12 %
**Morphology**	**28A-2^T**	**1**
Cell shape	Rods	Rods
Cell dimensions	2.7–3.5 x 0.6–1.0 µm	1.8–2.0 x 0.5–0.6 µm
Flagellated	Yes	Yes
Colony colour	Off-white	Cream-white

Table S8. The species most closely related to strain 28M-43^T based on NCBI BLAST identity of 16S rRNA gene sequences.

**BLAST relative**	**Identity**	**Query coverage***	**E value**
Psychrobacter nivimaris 88/2-7	99.93 %	97 %	0.0
Psychrobacter muriicola 2pS[†]	99.80 %	96 %	0.0
Psychrobacter adeliensis DSM 15333	99.61 %	99 %	0.0
Psychrobacter proteolyticus 116	99.37 %	92 %	0.0
Psychrobacter aquimaris SW-210	99.26 %	97 %	0.0
Psychrobacter piscatorii T-3-2	99.20 %	98 %	0.0

Psychrobacter aquaticus CMS 56	98.99 %	96 %	0.0
Psychrobacter namhaensis SW-242	98.86 %	97 %	0.0
Psychrobacter cryohalolentis K5	98.83 %	100 %	0.0

*All partial sequences, hence, coverage < 100 %. †Not a validated species.

Table S9. Phylogenomics of closely related strains of 28M-43^T based on digital DNA-DNA hybridization (dDDH) and average nucleotide identity (ANI). Strains with dDDH values below 70 % and ANI values below 95 % can be considered as separate species to strain 28M-43^T.

**Strain**	**dDDH (%)**	**ANI (%)**	**% G+C difference**
Psychrobacter nivimaris 88-2-7	54.6	94.3	0.19
Psychrobacter proteolyticus HAMBI_2948	49.9	93.7	0.14
Psychrobacter piscatorii T-3-2	26.2	84.8	0.39
Psychrobacter pacificensis DSM 23406	25.0	84.2	1.02
Psychrobacter fjordensis BSw21516B	24.6	82.81	0.48
Psychrobacter fozii CECT 5889	24.5	82.71	0.59

Table S10. A comparison of strain 28M-43^T and its closest relatives based on phenotypic and physiological characterisation. Strains: 1, *Psychrobacter adeliensis* SJ 14^T; 2, *Psychrobacter nivimaris* 88/2-7^T; 3, *Psychrobacter proteolyticus* 116^T. The data for *P. adeliensis*, *P. nivimaris*, and *P. proteolyticus* were taken from the literature. Growth conditions were tested on unbuffered nutrient agar. API 20NE strips were used to determine the enzymatic activities of 28M-43^T and its ability to grow with different substrates as sole carbon sources. A positive

result is indicated by '+', a negative result by '-', a weakly positive result by 'w', and no data by 'ND'.

**Enzymatic activities**	**28M-43^T**	**1**	**2**	**3**
Reduction of nitrates to nitrites	-	+	ND	-
Reduction of nitrates to dinitrogen	-	-	ND	-
Indole production	-	-	ND	-
Fermentation	-	-	ND	+
Arginine dihydrolase	-	-	ND	-
Urease	-	-	ND	+
Aesculin hydrolysis	-	-	ND	-
Gelatinase	-	-	-	+
β-Galactosidase	-	-	ND	-
Oxidase	+	+	+	+
**Growth substrates**	**28M-43^T**	**1**	**2**	**3**
D-Glucose	-	-	w	ND
L-Arabinose	-	-	w	ND
D-Mannose	-	-	w	ND
D-Mannitol	-	-	ND	ND
N-Acetylglucosamine	-	ND	ND	ND
D-Maltose	-	-	ND	ND
Gluconic acid	-	ND	ND	ND
Capric acid	-	ND	ND	-
Adipic acid	-	ND	ND	-
Malic acid	-	ND	-	-
Citric acid	-	-	-	+
Phenylacetic acid	-	ND	ND	-

**Growth conditions**	**28M-43^T**	**1**	**2**	**3**
Temperature range	4–34 °C	2–30 °C	5–35 °C	ND
pH range (unbuffered)	5–12	6–9	5.5–10	ND
Salinity (w/v NaCl)	0–12 %	0–10 %	0–13 %	ND
**Morphology**	**28M-43^T**	**1**	**2**	**3**
Cell shape	Cocci	Cocci	Coccobacilli	Coccobacilli
Cell dimensions	1.2–1.6 µm	ND	0.8–1.0 x 1.7–2.2	0.5–1.25 x 1.0–2.5 µm
Flagellated?	No	No	No	No
Colony colour	Cream	ND	Cream	Cream

Table S11. OSMAC screen for the antibacterial activity of strain 28M-7^T against *S. aureus* Newman and *A. baumannii*. A tick indicates that the strain was able to inhibit the growth of overlaid bacteria after growing on that agar at 28 °C for a week. Strain 28M-43^T was inactive against both strains on all the OSMAC media.

**Carbon source added to M9 agar**	**Inhibition of:**	
	***S. aureus* Newman**	***A. baumannii***
10 g/l dextrose	-	-
2 g/l dextrose	-	-
10 g/l mannitol	-	-
2 g/l mannitol	-	-
10 g/l glycerol	-	-
2 g/l glycerol	-	-
**10 g/l succinate**	✓	**-**
2 g/l succinate	-	-
10 g/l soluble starch	-	-
2 g/l soluble starch	-	-
10 g/l colloidal chitin	-	-

2 g/l colloidal chitin	-	-
No carbon source	-	-

Table S12. Comparative ^{1}H NMR spectroscopic data for the 28M-7ᵀ metabolite in $CDCl_3$, compared to the published values for agrochelin and massiliachelin in the same solvent.

[1]H Comparison

	**Massiliachelin**	**Agrochelin**	**28M-7ᵀ metabolite**
**1**			
**2**			
**3**			
**4**	6.76 (d 7.4)	6.70 (d, 7.8)	6.73 (d, 7.5)
**5**	7.33 (t, 8.0)	7.20 (t, 7.8)	7.26 (t, 7.5)
**6**	7.22 (d, 8.5)	6.84 (d, 7.8)	6.96 (br s)
**7**	2.92 (ddd, 14.4, 9.8, 6.4), 2.76 (14.4, 9.8, 6.3)	2.93 (m)	2.97 (m), 2.90 (m)
**8**	1.58 (m)	1.60 (m)	1.61 (m)
**9**	1.33 (m)	1.33 (m)	1.35 (overlapping)
**10**	1.33 (m)	1.33 (m)	1.35 (overlapping)
**11**	0.89 (t, 7.1)	0.89 (t, 6.9)	0.90 (t, 7.0)
**12**			
**13**	3.72 (dd, 12.1, 9.9), 3.46 (dd, 12.1, 6.4)	3.46 (dd, 11.3, 8.7), 3.14 (dd 11.3, 8.7)	3.52 (br t, 10.5), 3.22 (dd, 11.5, 7.0)
**14**	5.09 (td, 9.9, 6.4)	4.72 (q 8.7)	4.77 (m)
**15**	4.20 (d, 9.9)	4.18 (d, 9.3)	4.20 (d 9.0)
**16**	3.23 (d, 7.9)	3.27 (dd, 11.6, 7.2), 2.93 (m)	3.33 (m) and 3.03 (m)
**17**	3.49 (dt, 7.9, 2.6)	3.37 (m)	3.33 (overlapping m)
**18**	2.66 (s)	2.62 (s)	2.65 (s)
**19**	3.31 (d, 2.6)	3.49 (d, 7.0)	3.39 (d, 6.0)
**20**			
**21**	1.38 (s)	1.28 (s)	1.35 (overlapping)

**22**	1.25 (s)	1.25 (s)	1.23 (s)

Table S13. Comparative ^{13}C NMR spectroscopic data for the 28M-7^{T} metabolite in $CDCl_3$, compared to the published values for agrochelin and massiliachelin in the same solvent.

^{13}C Comparison

Massiliachelin	Assignment	Agrochelin	Assignment	28M-7^{T} Metabolite	Assignment
**182.3**	C-12	180	C23	183.4	C12
**181.3**	C-23	172.1	C12	178	C23
**158.8**	C1	159.6	C1	159.5	C1
**144.7**	C3	143.7	C3	144	C3
**135.9**	C5	132.2	C5	133	C5
**122.2**	C4	121.4	C4	121.8	C4
**116.1**	C6	116.1	C2	115.7	C2 or C6
**112.6**	C2	115.4	C6	115.5	C2 or C6
**80.4**	C15	79.3	C15	79.5	C15
**78.6**	C19	77.8	C14	78	C19
**73.1**	C17	77	C19	76.5 (obscured)	C14
**70.7**	C14	73	C17	73.3	C17
**47.3**	C18	46.2	C18	46.7	C18
**44.3**	C20	46	C20	45.6	C20
**36.1**	C16	36.5	C16	36.5	C16
**35.1**	C7	35.3	C7	35.4	C7
**33.5**	C13	35	C13	34.8	C13
**31.8**	C8	32.1	C8	32.3	C8
**31.7**	C9	31.8	C9	31.9	C9
**26.5**	C21	22.4	C10	24.5	C21/22
**22.4**	C10	22.1	C21	22.5	C10
**22.1**	C22	22	C22	21.9	C21/22

**14**	C11	14	C11	14.1	C11

Table S14. Strain 28M-7^T thiazole alkaloid BGC as identified by antiSMASH. BGC boundaries are shown as black lines. GenBank protein accession numbers for the minimal agrochelin BGC are given beneath the gene names.

**ORF**	**Size (aa)**	**Predicted function**	**Closest BLAST relative (% identity, % coverage)**
orf -10	332	Oxidoreductase	Gfo family oxidoreductase<br>*Stappia* sp. ARW1T (100, 100)
orf -9	482	Transcription factor	MocR family regulator<br>*Stappia* sp. ARW1T (98, 100)
orf -8	286	Transporter	DMT family transporter<br>*Stappia* sp. ARW1T (99, 100)
orf -7	293	Transcription factor	LysR family regulator<br>*Stappia indica* (99, 100)
orf -6	499	Methylmalonate-semialdehyde dehydrogenase	CoA-acylating methylmalonate-semialdehyde dehydrogenase<br>*Stappia indica* (100, 100)
orf -5	397	Acetyl-CoA C-acyltransferase	Acetyl-CoA C-acyltransferase<br>*Stappia* sp. ARW1T (100, 100)
orf -4	335	Haem-binding or iron-regulated lipoprotein	ChaN family lipoprotein<br>*Stappia* sp. ARW1T (96, 100)
orf -3	108	Unknown	Hypothetical protein<br>*Stappia* sp. ARW1T (100, 100)
orf -2	705	K^+/H^+ antiporter	Potassium/proton antiporter<br>*Stappia* sp. ARW1T (100, 100)
orf -1	376	NADP-dependent dehydrogenase	Saccharopine dehydrogenase NADP-binding protein

			Stappia sp. ARW1T (100, 100)
agrR *MBC2859116.1*	334	Transcription factor	HTH transcriptional regulator *Stappia* sp. ARW1T (100, 100)
agrA *MBC2859117.1*	4193	Agrochelin synthase 1 NRPS/PKS	Amino acid adenylation domain-containing protein *Stappia* sp. ARW1T (98, 100)
agrB *MBC2859118.1*	2219	Agrochelin synthase 2 NRPS/PKS	NAD(P)-dependent oxidoreductase *Stappia* sp. ARW1T (97, 100)
agrC *MBC2859119.1*	374	Oxidoreductase	Gfo family oxidoreductase *Stappia* sp. ARW1T (99, 100)
agrD *MBC2859120.1*	546	Terminating thioesterase and/or methyltransferase	Alpha/beta fold hydrolase *Stappia* sp. ARW1T (98, 100)
agrE *MBC2859121.1*	256	Proofreading thioesterase	Thioesterase *Stappia* sp. ARW1T (98, 100)
agrT1 *MBC2859122.1*	421	Acetyl-CoA transporter	MFS transporter *Stappia* sp. ARW1T (99, 100)
agrT2 *MBC2859123.1*	672	Agrochelin-Fe importer	TonB-dependent receptor *Stappia* sp. ARW1T (99, 100)
agrF *MBC2859124.1*	384	Oxidoreductase	FAD-dependent monooxygenase *Stappia* sp. ARW1T (99, 100)
orf +1	137	Transcription factor	Cu(I)-responsive transcription regulator *Stappia* sp. ARW1T (99, 100)
orf +2	139	Cytochrome c	Cytochrome c *Stappia* sp. ARW1T (99, 99)
orf +3	240	Transporter	ABC transporter ATP-binding protein

			Stappia sp. ARW1T (99, 100)
orf +4	266	Transporter	ABC transporter ATP-binding protein<br>*Stappia indica* (99, 100)
orf +5	315	Transporter	Branched-chain amino acid ABC transporter permease<br>*Stappia* sp. ARW1T (99, 100)
orf +6	290	Transporter	Branched-chain amino acid ABC transporter permease<br>*Stappia indica* (100, 100)
orf +7	384	Transporter	ABC transporter substrate-binding protein<br>*Stappia* sp. ARW1T (100, 100)
orf +8	553	Aldehyde dehydrogenase (phenylacetic acid degradation)	Phenylacetic acid degradation protein PaaN<br>*Stappia* sp. ARW1T (99, 100)
orf +9	287	Enoyl-CoA hydratase<br>(phenylacetic acid degradation)	2-(1,2-Epoxy-1,2-dihydrophenyl)<br>acetyl-CoA isomerase<br>*Stappia indica* (99, 98)
orf +10	154	Thioesterase<br>(phenylacetic acid degradation)	Acyl-CoA thioesterase<br>*Stappia* sp. ARW1T (98, 100)
orf +11	436	Phenylacetate-CoA ligase<br>(phenylacetic acid degradation)	Phenylacetate-CoA ligase<br>*Stappia* sp. ARW1T (100, 100)
orf +12	200	Transcription factor	TetR family transcription regulator *Stappia* sp. ARW1T (100, 100)

orf +13	493	Nucleoid-associated protein	LysM domain-containing protein *Stappia* sp. ARW1T (99, 100)
orf +14	213	Lysine decarboxylase	TIGR00730 family protein<br>*Stappia indica* (97, 100)
orf +15	1203	Efflux pump	Efflux RND transporter permease subunit<br>*Stappia* sp. ARW1T (100, 100)
orf +16	367	Efflux pump	Efflux RND transporter periplasmic adaptor subunit<br>*Stappia* sp. ARW1T (100, 100)
orf +17	184	Transcription factor	HTH transcriptional regulator<br>*Stappia* sp. ARW1T (100, 100)
orf +18	126	Unknown	Hypothetical protein<br>*Stappia* sp. ARW1T (100, 100)
orf +19	303	Allantoinase	Allantoinase PuuE<br>*Stappia* sp. ARW1T (100, 100)
orf +20	321	Transcription factor<br>(phenylacetic acid degradation)	Phenylacetic acid degradation<br>protein PaaX<br>*Stappia* sp. ARW1T (99, 100)
orf +21	333	PaaA<br>(phenylacetic acid degradation)	1,2-Phenylacetyl-CoA<br>epoxidase subunit A<br>*Stappia* sp. ARW1T (99, 100)
orf +22	95	PaaB<br>(phenylacetic acid degradation)	1,2-Phenylacetyl-CoA<br>epoxidase subunit B<br>*Stappia indica* (99, 100)
orf +23	255	PaaC	Phenylacetate-CoA

		(phenylacetic acid degradation)	oxygenase subunit PaaC<br>*Stappia* sp. ARW1T (100, 100)
orf +24	172	PaaD<br>Metal-sulphur cluster biosynthetic enzyme<br>(phenylacetic acid degradation)	Phenylacetate-CoA<br>oxygenase subunit PaaJ<br>*Stappia* sp. ARW1T (100, 98)
orf +25	359	PaaK<br>(phenylacetic acid degradation)	Phenylacetate-CoA oxygenase/<br>reductase subunit PaaK<br>*Stappia* sp. ARW1T (100, 100)
orf +26	181	ATPase	AAA family ATPase<br>*Stappia indica* (91, 100)
orf +27	339	Alcohol dehydrogenase	Zn-dependent alcohol dehydrogenase<br>*Stappia* sp. ARW1T (100, 100)
orf +28	495	Diguanylate cyclase	Diguanylate cyclase<br>*Stappia* sp. ARW1T (99, 100)
orf +29	130	Vicinal oxygen chelate protein<br>(phenylacetic acid degradation)	VOC family protein<br>*Stappia* sp. ARW1T (99, 100)

Table S15. Genome accessions and GTDB species identification used for *Stappia* BiG-SCAPE analysis.

**Accession**	**Strain / label (NCBI)**	**GTDB genus / species**	**GTDB species rep?**
GCA_002684215.1	*Stappia* sp. CPC38 (MAG)	*Stappia* sp002722295	No
GCA_002709505.1	Rhodobacteraceae bacterium (MAG)	*Stappia* sp002709505	Yes

GCA_002722295.1	*Stappia* sp. SP245 (MAG)	*Stappia* sp002722295	Yes
GCA_016774605.1	Alphaproteobacteria bacterium (MAG)	*Stappia* sp016774605	Yes
GCF_000423705.1	*Stappia stellulata* DSM 5886	*Stappia stellulata*	Yes
GCF_001696535.1	*Stappia indica* SBBC 49	*Stappia indica*	Yes
GCF_001696545.1	*Stappia indica* EBBD 17.2	*Stappia indica*	No
GCF_003610575.1	*Stappia* sp. ARW1T	*Stappia* sp003610575	Yes
GCF_009789575.1	*Stappia indica* PHM037	*Stappia indica_A*	Yes
GCF_013868145.1	*Stappia taiwanensis*	*Stappia taiwanensis*	No
GCF_014252955.1	*Stappia* sp. 28M-7	*Stappia* sp003610575	No
GCF_014635285.1	*Stappia taiwanensis*	*Stappia taiwanensis*	Yes
GCF_900105595.1	*Stappia* sp. ES.058	*Stappia* sp900105595	Yes
GCF_900185725.1	*Stappia* sp. TSB10P1A	*Stappia* sp900185725	Yes
GCF_900185735.1	*Stappia* sp. P2PMeth1	*Stappia* sp900185725	No
GCF_900185745.1	*Stappia* sp. TSB10GB4	*Stappia* sp900185725	No
GCF_900217825.1	*Stappia indica* USBA 352	*Stappia indica*	No

Table S16. Media used in this study.

**Component**	**Amount per litre**
**Marine agar**	
Marine agar (Difco)	37.4 g
Agar	15 g
**M9 10 g/l succinate broth (adjust pH to 7.0)**	
5x M9 salts (Serva)	10.44 g
1 M $MgSO_4$	2 ml
1 M $CaCl_2$	0.1 ml
Succinate	10 g
0.5 % w/v Thiamine mononitrate	0.1 ml
Casamino acids (Bio Basic Inc)	1 g
**Mueller Hinton agar**	
Mueller Hinton broth (Sigma)	22 g

Agar	15 g
**AIA agar**	
Actinomycetes isolation agar (Sigma)	22 g
**Nutrient Agar (adjusted to pH 7.0)**	
Peptone	5 g
Yeast extract	3 g
NaCl	5 g
Agar	15 g
**Luria-Bertani (LB) agar**	
Tryptone	10 g
Yeast extract	5 g
NaCl	10 g
Agar	15 g

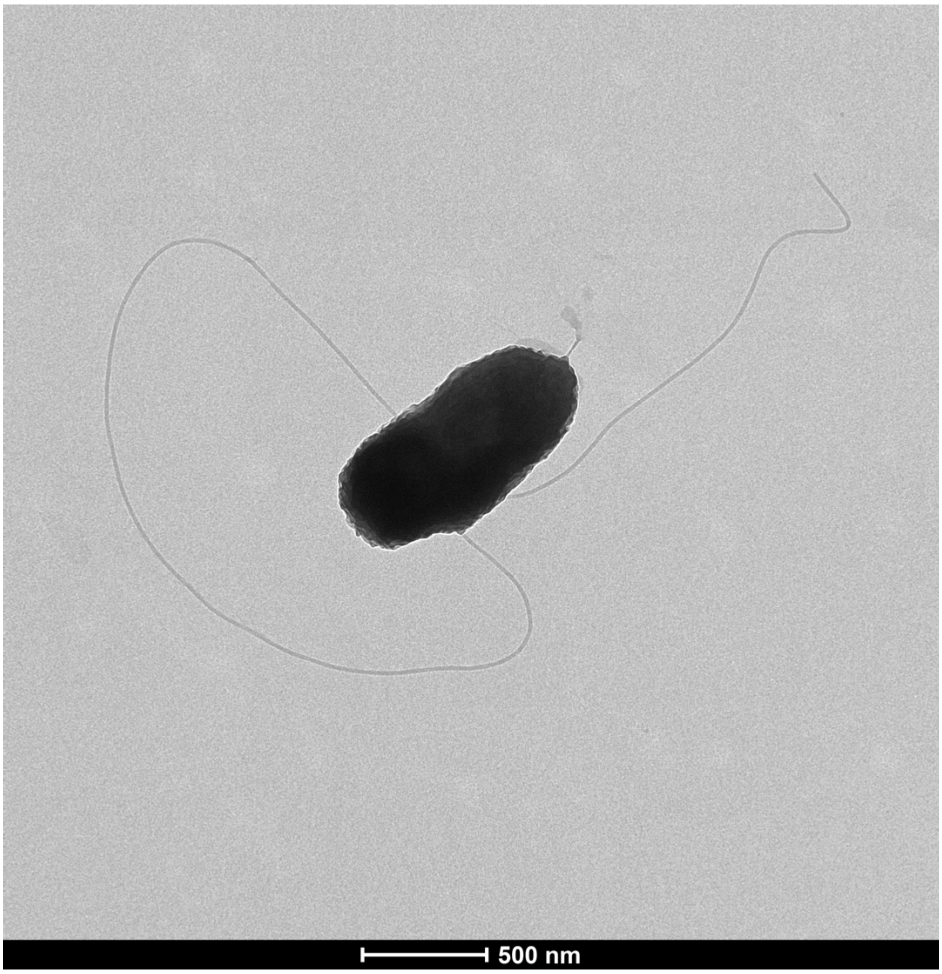

Figure S1. Transmission electron micrograph of 28M-7^{T}.

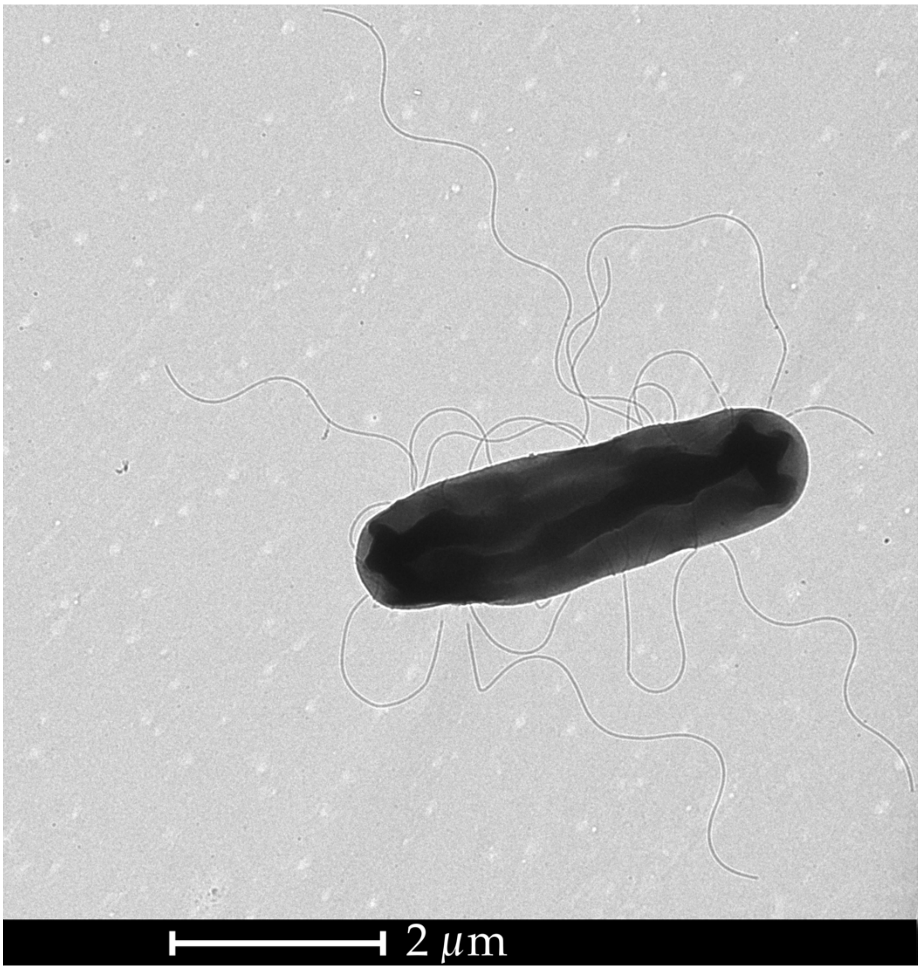

Figure S2. Transmission electron micrograph of 28A-2^T.

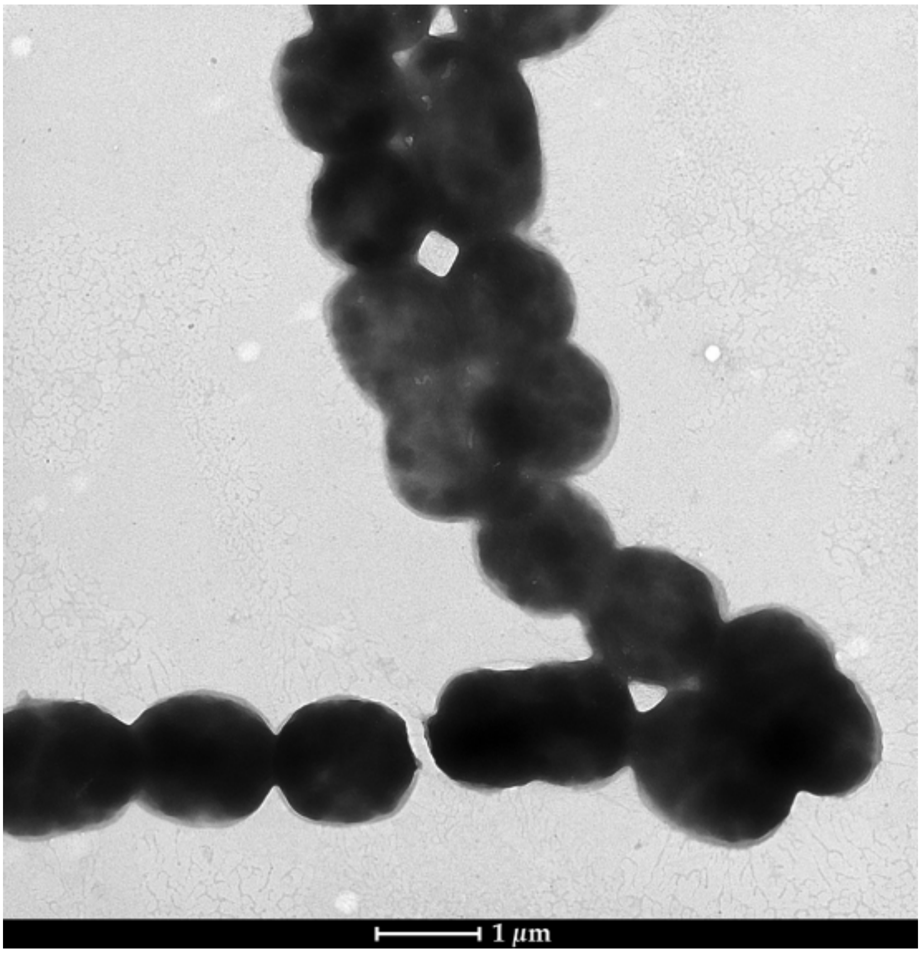

Figure S3. Transmission electron micrograph of 28M-43^{T}.

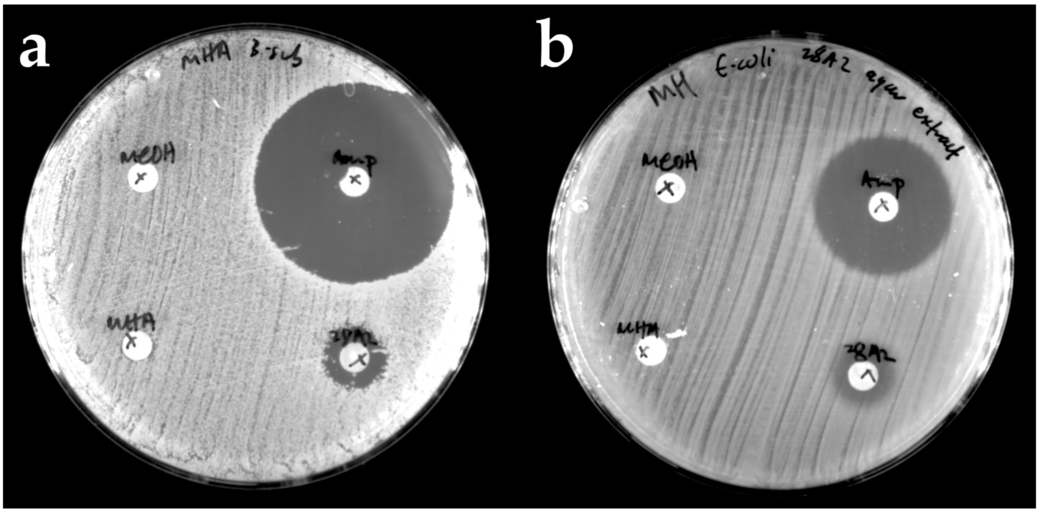

Figure S4. Extracts of strain 28A-2ᵀ grown on solid media screened for antibacterial activity against (a) *B. subtilis* and (b) *E. coli*. For each plate, 10 μl of the following was applied to each paper disc, clockwise from top left: methanol; 100 mg/ml ampicillin in water; an ethyl acetate extract of 28A-2ᵀ agar plates redissolved in methanol; an ethyl acetate extract of sterile Mueller-Hinton agar plates redissolved in methanol.

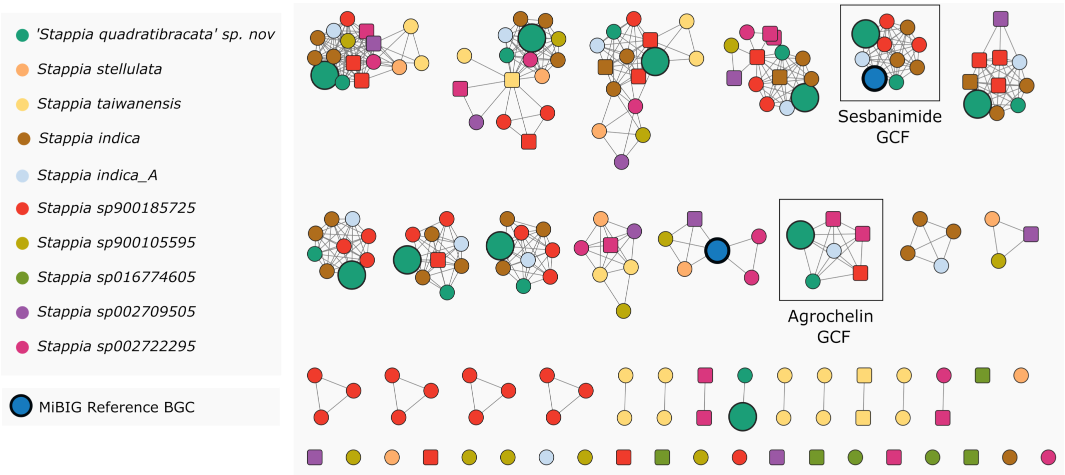

Figure S5. Gene cluster family analysis of seventeen genomes from the *Stappia* genus using BiG-SCAPE2. BGCs from MiBIG 4.0 are highlighted in blue. Nodes are coloured according to GTDB species assignment and edges represent BiG-SCAPE distance score with cut-off set to 0.35. BGC nodes from strain 28M7ᵀ are enlarged. BGC nodes located on a contig edge, potentially fragmented, are rectangular.

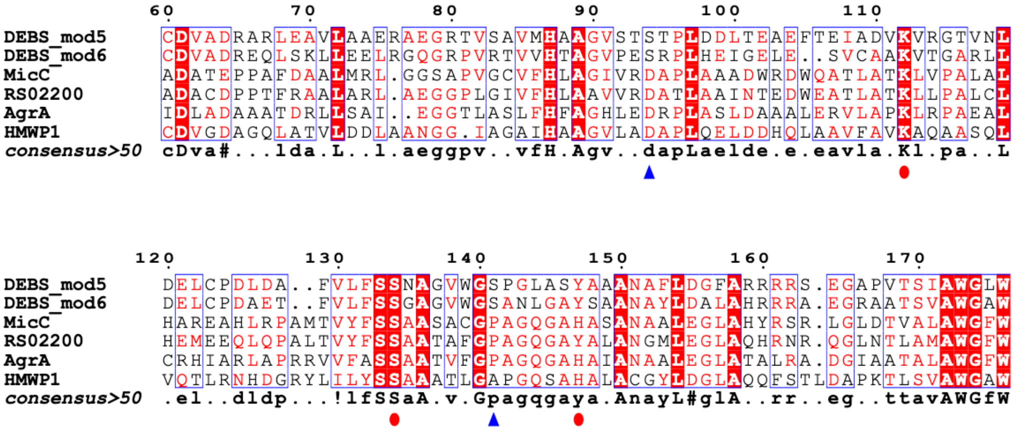

Figure S6. Alignment of the ketoreductase domains of DEBS modules 5 & 6 (Erythromycin), MicC (Micacocidin), RS02200 (Massiliachelin), AgrA (this study), and HMWP1 (yersinibactin). The red circles show the catalytic residues Lys112, Ser134, Tyr147. Blue triangles show Asp94, and Pro141, residues associated with B-type alcohol stereochemistry.

Compound Characterisation; Agrochelin II

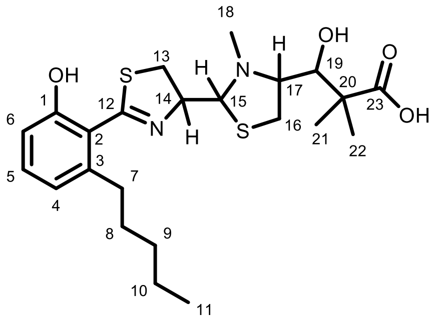

δ_H (600 MHz, $CDCl_3$) 7.26 (1H, t, *J* 7.5, 5-H), 6.96 (1H, br s, H-6), 6.73 (1H, d, *J* 7.5, 4-H), 4.77 (1H, q, *J* 8.5,14-H), 4.20 (1H, d, *J* 9.0, 15-H), 3.52 (1H, t, *J* 10.0,13-*H*H), 3.39 (1H, d, *J* 6.0, 19-H), 3.36 – 3.28 (2H, overlapping m, 16-*H*H and 17-H), 3.22 (1H, dd, *J* 11.5, 7.0, 13-H*H*), 3.03 – 3.00 (1H, m, 16-H*H*), 2.99 – 2.95 (1H, m, 7-H*H*), 2.92 – 2.87 (1H, m, 7-*H*H), 2.65 (3H, s,18-H_3), 1.61 (2H, m, 8-H_2), 1.40 –1.31 (7H, m, 22-H_3, 9-H_2 and 10-H_2), 1.23 (3H, s, 21-H_3), 0.90 (3H, t, *J* 7.0, 11-H_3). δ_C (150 MHz, $CDCl_3$) 183.4 (C-12), 178.0 (C-23), 159.5 (C-1), 144.0 (C-3), 133.0 (C-5), 121.8 (C-4), 115.7 (C-2/C-6), 115.5 (C-2/C-6), 79.5 (C-15), 78.0 (C-19), 76.8 (obscured, C-14), 73.4 (C-17), 46.7 (C-18), 45.6 (C-20), 36.5 (C-16), 35.4 (C-7), 34.8 (C-13), 32.3 (C-8), 31.9 (C-9), 24.5 (C-21/22), 22.5 (C-10), 21.9 (C-21/22) 14.1 (C-11).

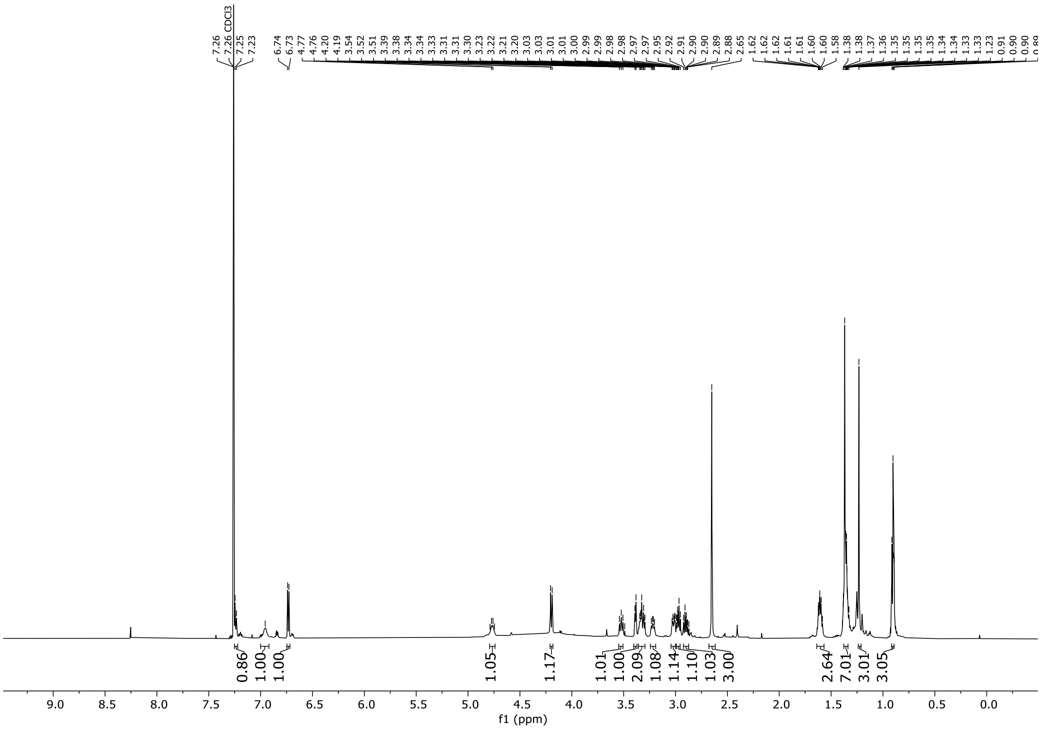
7.26
7.26 CDCl3
7.25
7.23
6.74
6.73
4.77
4.76
4.20
4.19
3.54
3.52
3.51
3.39
3.38
3.34
3.34
3.33
3.31
3.31
3.30
3.23
3.22
3.21
3.20
3.03
3.03
3.01
3.01
3.00
2.99
2.99
2.98
2.98
2.97
2.97
2.97
2.95
2.92
2.91
2.90
2.90
2.89
2.88
2.65
1.62
1.62
1.61
1.61
1.60
1.60
1.58
1.38
1.38
1.37
1.36
1.35
1.35
1.35
1.35
1.34
1.34
1.33
1.33
1.23
0.91
0.90
0.86
1.00
1.00
1.05
1.17
1.01
1.00
2.09
1.08
1.14
1.10
1.03
3.00
2.64
7.01
3.01
3.05
9.0 8.5 8.0 7.5 7.0 6.5 6.0 5.5 5.0 4.5 4.0 3.5 3.0 2.5 2.0 1.5 1.0 0.5 0.0
f1 (ppm)

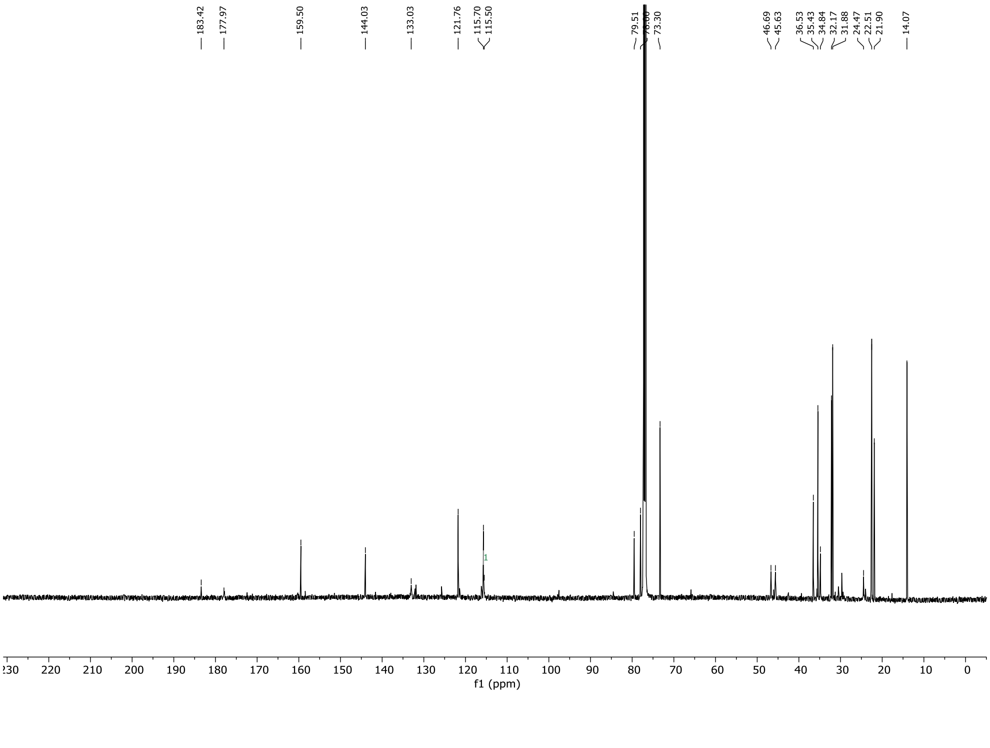
183.42
177.97
159.50
144.03
133.03
121.76
115.70
115.50
79.51
[illegible]
73.30
46.69
45.63
36.53
35.43
34.84
32.17
31.88
24.47
22.51
21.90
14.07
230 220 210 200 190 180 170 160 150 140 130 120 110 100 90 80 70 60 50 40 30 20 10 0
f1 (ppm)

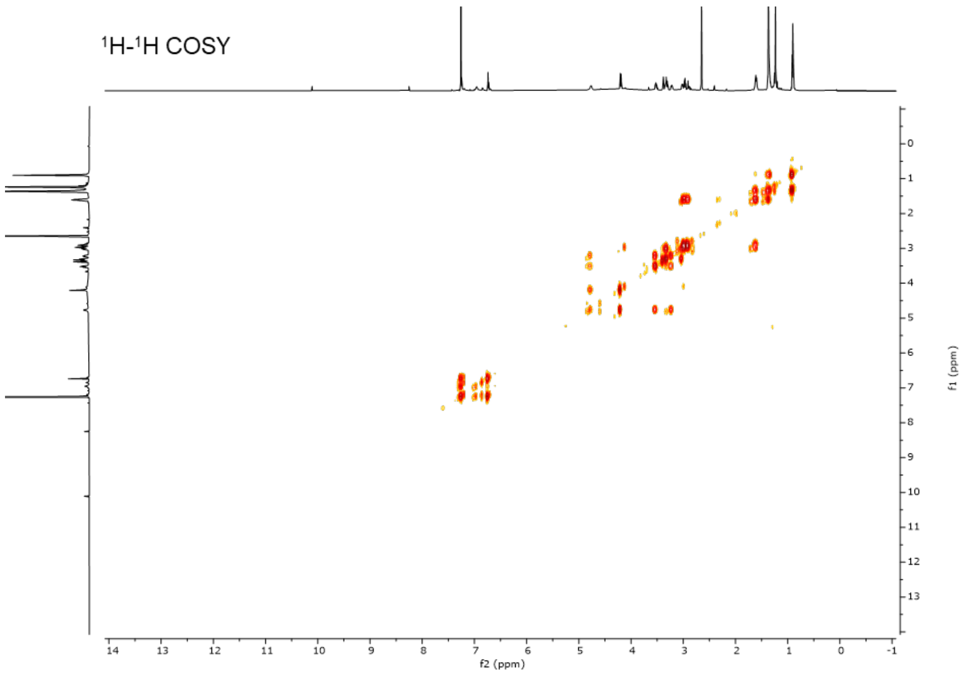

1H-1H COSY
f2 (ppm)
f1 (ppm)

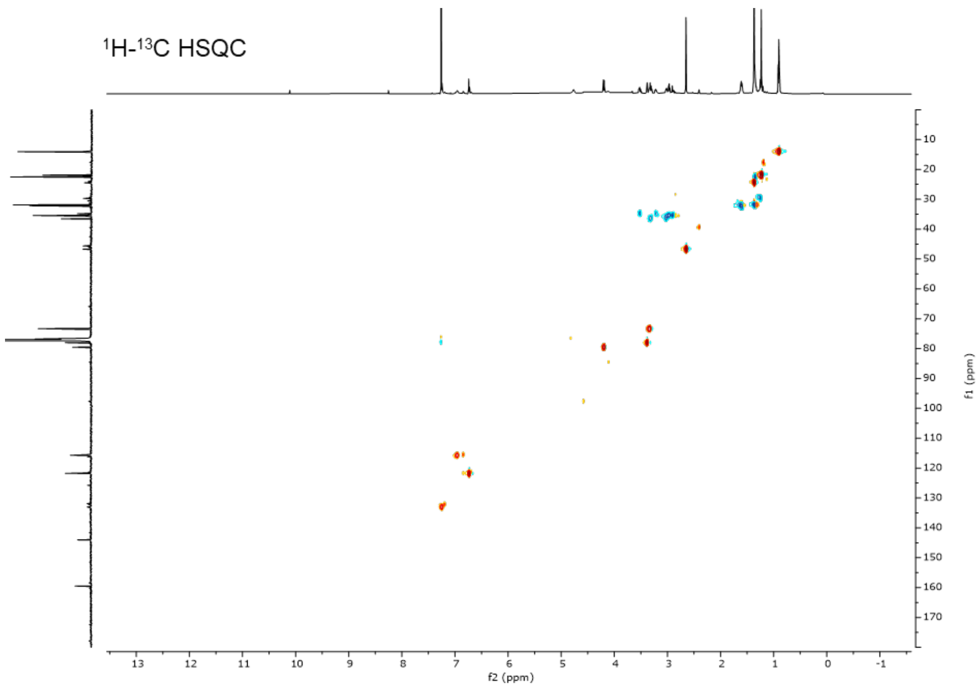

1H-13C HSQC
f2 (ppm)
f1 (ppm)

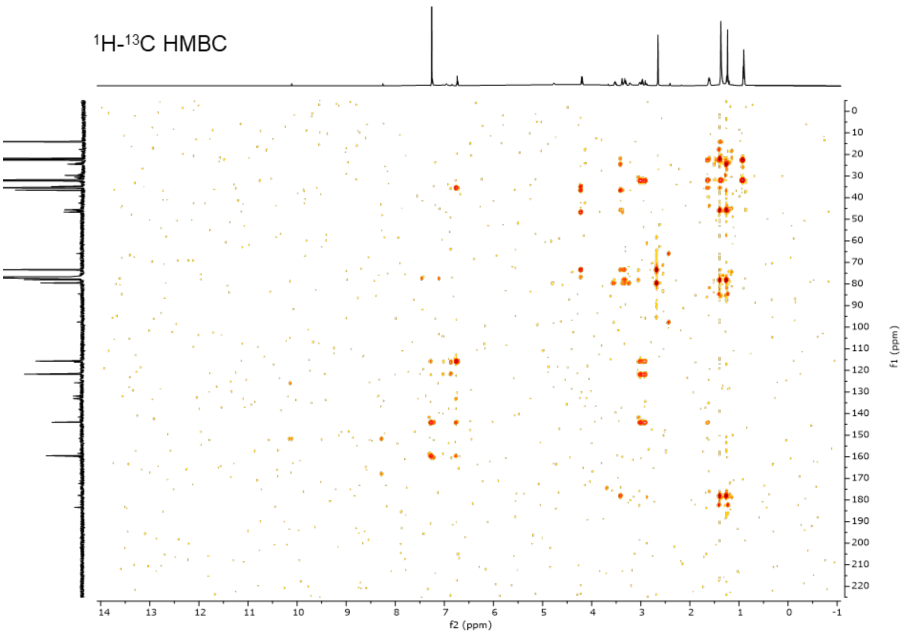

^1H-^{13}C HMBC
f2 (ppm)
f1 (ppm)

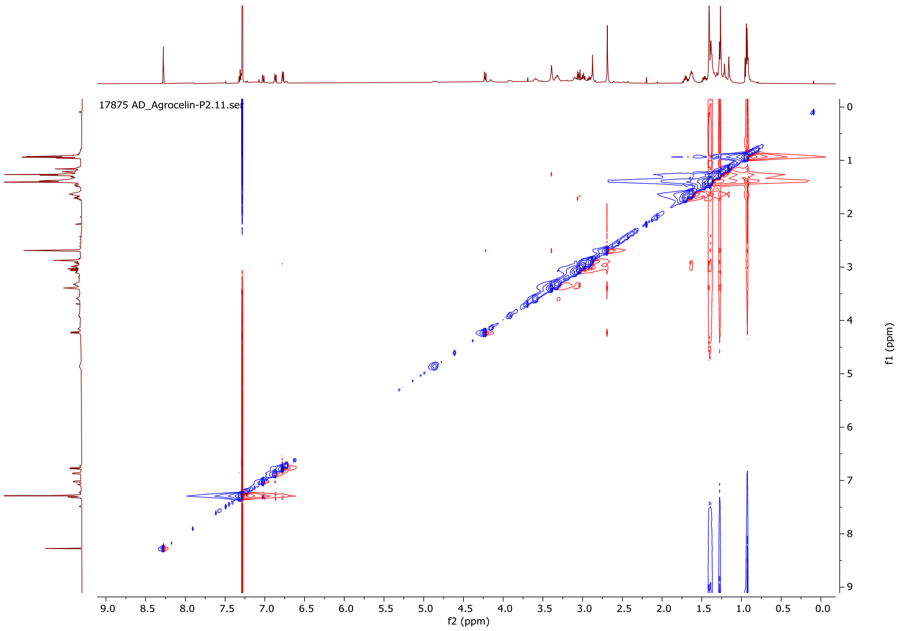

17875 AD_Agrocelin-P2.11.ser
f2 (ppm)
f1 (ppm)
